# Aberrant Mitochondrial Dynamics and Exacerbated Response to Neuroinflammation in a Novel Mouse Model of CMT2A

**DOI:** 10.3390/ijms222111569

**Published:** 2021-10-26

**Authors:** Filippos Stavropoulos, Irene Sargiannidou, Louiza Potamiti, Alexia Kagiava, Mihalis I. Panayiotidis, Ji Hyun Bae, Su Cheong Yeom, Jae Young Lee, Kleopas A. Kleopa

**Affiliations:** 1 Neuroscience Department, The Cyprus Institute of Neurology and Genetics, Nicosia 2371, Cyprus; filipposs@cing.ac.cy (F.S.); irenes@cing.ac.cy (I.S.); alexiak@cing.ac.cy (A.K.); 2 Department of Cancer Genetics, Therapeutics & Ultrastructural Pathology, The Cyprus Institute of Neurology and Genetics, Nicosia 2371, Cyprus; louizap@cing.ac.cy (L.P.); mihalisp@cing.ac.cy (M.I.P.); 3 Graduate School of International Agricultural Technology and Institute of GreenBio Science and Technology, Seoul National University, Pyeongchang 25354, Korea; jh_bae@kakao.com (J.H.B.); scyeom@snu.ac.kr (S.C.Y.); 4 ToolGen Inc., Seoul 08501, Korea; jy.lee2@toolgen.com; 5 Center for Neuromuscular Disorders and Center for Multiple Sclerosis and Related Disorders, The Cyprus Institute of Neurology and Genetics, Nicosia 2371, Cyprus

**Keywords:** Charcot-Marie-Tooth disease type 2A, peripheral neuropathy, knock-in mouse model, mitofusin-2, mitochondria, lipopolysaccharide, neuroinflammation

## Abstract

Charcot-Marie-Tooth disease type 2A (CMT2A) is the most common hereditary axonal neuropathy caused by mutations in *MFN2* encoding Mitofusin-2, a multifunctional protein located in the outer mitochondrial membrane. In order to study the effects of a novel *MFN2^K357T^* mutation associated with early onset, autosomal dominant severe CMT2A, we generated a knock-in mouse model. While *Mfn2^K357T/K357T^* mouse pups were postnatally lethal, *Mfn2^+/K357T^* heterozygous mice were asymptomatic and had no histopathological changes in their sciatic nerves up to 10 months of age. However, immunofluorescence analysis of *Mfn2^+/K357T^* mice revealed aberrant mitochondrial clustering in the sciatic nerves from 6 months of age, in optic nerves from 8 months, and in lumbar spinal cord white matter at 10 months, along with microglia activation. Ultrastructural analyses confirmed dysmorphic mitochondrial aggregates in sciatic and optic nerves. After exposure of 6-month-old mice to lipopolysaccharide, *Mfn2^+/K357T^* mice displayed a higher immune response, a more severe motor impairment, and increased CNS inflammation, microglia activation, and macrophage infiltrates. Overall, ubiquitous *Mfn2^K357T^* expression renders the CNS and peripheral nerves of *Mfn2^+/K357T^* mice more susceptible to mitochondrial clustering, and augments their response to inflammation, modeling some cellular mechanisms that may be relevant for the development of neuropathy in patients with CMT2A.

## 1. Introduction

Charcot-Marie-Tooth (CMT) disease is one of the most common inherited neurological disorders, with a varying prevalence of approximately 1/2500 individuals in Western Norway [1], 1/9804 in Western Japan [2], 1/8475 in Northern England [3], and 1/6369 in Auckland, New Zealand [4]. Due to genetic and clinical heterogeneity, CMT is divided into types and sub-types. From the axonal CMT types (CMT2), the most frequent sub-type is CMT2A caused by mutations, mostly dominant [5,6,7], in the *Mitofusin-2* (*MFN2*) gene [8,9].

Although CMT2A manifests typically as a length-dependent peripheral neuropathy involving the lower and upper extremities [5,6,7,10,11,12,13], the central nervous system (CNS) may also be affected, manifesting with optic atrophy or other brain abnormalities [6,11], while sensorineural hearing loss has additionally been described [6,7,11]. In rare cases, involvement of adipose tissue [12,13], mitochondrial myopathy [11], and the presence of dysmorphic features accompanied by severe global developmental delay [14] have also been reported. This broad CMT2A clinical spectrum, with primary involvement of the peripheral nervous system (PNS) and CNS, is explained by the ubiquitous *MFN2* expression (in neuronal and non-neuronal tissues) [15,16]. However, the reason why *MFN2* pathogenic variants preferentially target the nervous system, especially peripheral nerves with long axons, has yet to be elucidated, although some mechanisms have been proposed.

*MFN2* is a nuclear-encoded gene whose product is a dynamin-like GTPase embedded in the outer membrane of mitochondria mediating mitochondrial fusion [17,18], endoplasmic reticulum (ER)-mitochondria tethering [19,20], transport of axonal mitochondria [21], and mitophagy [18]. MFN2 consists of four protein domains: a GTPase domain, a first coiled-coil heptad-repeat (HR1) domain, a transmembrane (TM) domain, and a second coiled-coil heptad-repeat (HR2) domain, all of them separated by spacer sequences [22,23,24].

A 4-year-old boy was evaluated in our clinic with a severe, early-onset (by 2 years of age) neuropathy associated with a *de novo*, novel, missense mutation in exon 11 (RefSeq accession number: NM_014874.4) of the *MFN2* gene (located on chromosome 1 (Chr. 1)). The mutation is caused by an A>C nucleotide substitution at position 1070 (c.1070A>C) in the protein coding sequence (c. or CDS) of *MFN2*. This single nucleotide change results in the substitution of the positively charged lysine (K) for the polar (uncharged) threonine (T) at position 357 in the human MFN2 (hMFN2) protein sequence (p.K357T).

Although not located in any of the characterized protein domains, the affected amino acid is situated within the highly conserved region 3 (R3) of the spacer sequence between the GTPase and the HR1 domain [23]. K357 along with R95 form a hinge (hinge 2) that connects HR1 to the GTPase domain [24], and a bending via hinge 2 is required for hMFN2 to enter the fusion-promoting transition state after GTP hydrolysis (catalyzed by the GTPase domain), similarly to its hMFN1 paralog [24,25]. Thus, mutations in this critical hinge 2 region are expected to interfere with the fusogenic activity of *MFN2*.

A knock-in (KI) mouse model was created for the characterization of the *MFN2^K357T^* CMT2A-causing mutation. Wild type (WT; *Mfn2^+/+^*) and mice heterozygous for the *Mfn2^K357T^* mutation (*Mfn2^+/K357T^*) were studied up to 10 months (mos) of age. *Mfn2^+/K357T^* mice showed normal peripheral nerve function, but developed aberrant clustering of mitochondria with abnormal morphology in their sciatic and optic nerves. Based on evidence of a neuroprotective and anti-inflammatory role of neuronally-expressed *MFN2* under lipopolysaccharide (LPS)-induced neuroinflammation [26], *Mfn2^+/+^* and *Mfn2^+/K357T^* mice were LPS challenged to reveal the manifestation of *Mfn2^K357T^* dominant effects. LPS triggered an exacerbated peripheral inflammatory response accompanied by increased CNS infiltration and microglial activation in *Mfn2^+/K357T^* mice, leading to more severe motor dysfunction compared to controls.

## 2. Results

### 2.1. Replication of the Human MFN2^K357T^ Mutation in a Mfn2^K357T^-Expressing CMT2A KI Mouse Model

**Figure 1:**
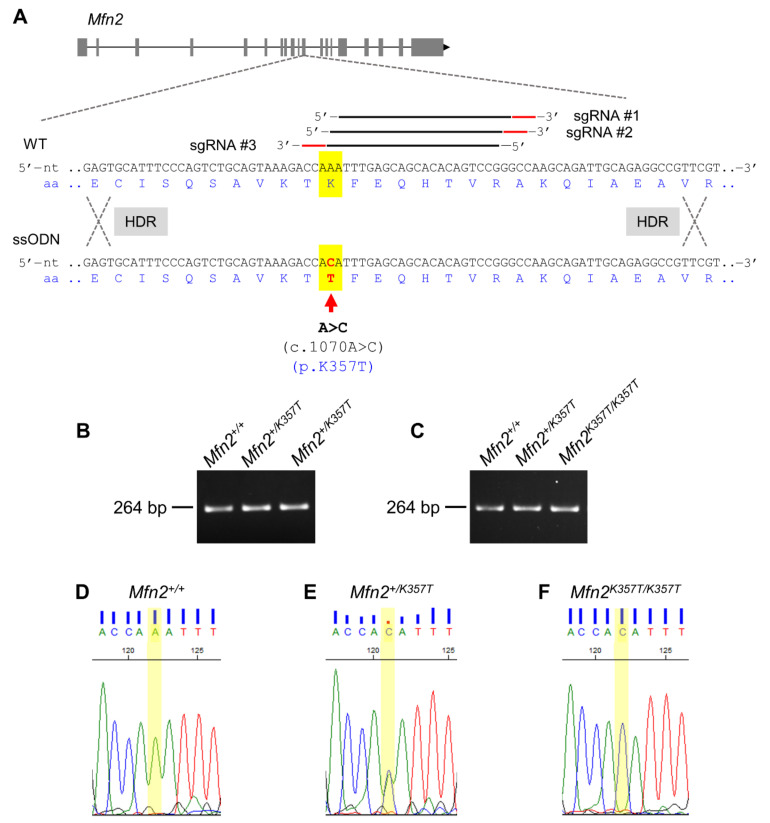
Generation and genotyping of the *Mfn2^+/K357T^* knock–in (KI) CMT2A mouse model. (**A**): The KI mouse model was developed by ssODN–mediated KI by CRISPR/Cas9. The mouse *Mfn2* gene located on chromosome 4 is illustrated with its 19 exons (grey boxes) and introns (black horizontal lines). The mutation was introduced in exon 11. A number of overlapping single guide RNAs (sgRNAs), consisting of a binding site (black horizontal line) and a protospacer–adjacent motif (PAM) sequence (red horizontal line), were developed to guide Cas9 to introduce double–strand breaks (DSBs) in the target sequence. The lysine (K) coding nucleotide triplet (AAA) in the WT sequence containing the nucleotide residue aimed for the A>C CMT2A causing substitution is highlighted in yellow. After the introduction of the DSBs, the synthetic ssODN carrying the A>C threonine (T) coding substitution (red arrow) is introduced to the mouse *Mfn2* locus via the HDR repair mechanism (nt: nucleotide; aa: amino acid). (**B**,**C**): Electrophoresis of the *Mfn2* PCR amplicon containing the region with the A>C nucleotide substitution from the tails of juvenile mice (**B**) and toes of mouse pups (**C**). (**D**–**F**): Representative sequencing electropherograms from a WT (*Mfn2^+/+^*, (**D**), heterozygous (*Mfn2^+/K357T^*, (**E**), and homozygous (*Mfn2^K357T/K357T^*, (**F**) mouse, depicting part of the *Mfn2* nucleotide sequence bearing the nucleotide residue affected (highlighted in yellow) in the event of the A>C substitution.

The human *MFN2* (CCDS ID: CCDS30587.1) and mouse *Mfn2* (CCDS ID: CCDS38965.1) (each 2274 nucleotides (nt) CDS; 757 aa) are 90% and 95% identical at their CDS and protein sequence, respectively. This high interspecies conservation, especially at the protein sequence, permits the replication of human CMT2A mutations in mice with high fidelity. The novel *MFN2^K357T^* CMT2A-causing mutation found in the CMT2A patient was introduced into the mouse endogenous *Mfn2* sequence (Chr.4, exon 11 (RefSeq accession number: NM_001285922.1)), following the ssODN-mediated KI with CRISPR-Cas approach (Figure 1A). The expression of the mutant *Mfn2* allele (*Mfn2^K357T^*) in the CMT2A KI mouse model is regulated by the mouse *Mfn2* promoter allowing for ubiquitous expression, as is the case with the widespread expression of *MFN2* mutations in CMT2A patients. For genotyping purposes, the *Mfn2* region containing the c.1070A>C substitution was PCR amplified from isolated DNA from the tails of juvenile mice (Figure 1B), or the toes of mouse pups (Figure 1C). The resultant PCR amplicon (~264 bp) was then Sanger-based sequenced to determine the genotype (Figure 1D).

### 2.2. Mfn2^K357T/K357T^ Homozygous Mouse Pups Are Susceptible to Postnatal Lethality and Present with Severe Mitochondrial Clustering

*Mfn2^+/+^* (WT), *Mfn2^+/K357T^* (heterozygous), and *Mfn2^K357T/K357T^* (homozygous) mouse pups were born at an approximate 1:2:1 Mendelian ratio (20:30:15, *Mfn2^+/+^*:*Mfn2^+/K357T^*:*Mfn2^K357T/K357T^*, with a chi-square *p*-value between 0.5 and 0.75). *Mfn2^+/K35T7^* and *Mfn2^K357T/K357T^* had a normal appearance and were phenotypically indiscernible at birth when compared to *Mfn2^+/+^* mouse pups; however, *Mfn2^K357T/K357T^* mouse pups tended to die hours or days after birth. Phenotypic manifestations in *Mfn2^K357T/K357T^* mouse pups began to emerge only in animals that died at later postnatal stages (P4–P8) and presented with stunted growth caused by developmental delay (Figure S1A). When checked every 24 h after birth, only 50% of *Mfn2^K357T/K357T^* mouse pups survived past P0 (0–24 h) and just 7.14% reached P8 (192–216 h) (Figure S1B).

Spinal cords from *Mfn2^+/+^*, *Mfn2^+/K35T7^*, *Mfn2^K357T/K357T^* mouse pups were stained for voltage-dependent anion channel 1 (VDAC1, a protein located in the outer mitochondrial membrane) (Figure S1C). Intense VDAC1^+^ fluorescent clusters were spotted in *Mfn2^K357T/K357T^* mouse pups, suggestive of aberrant mitochondrial clustering, a characteristic finding in the majority of *MFN2* mutations [22,27,28,29,30]. Assessment of VDAC1 total fluorescence intensity revealed a higher burden of VDAC1^+^ mitochondrial clusters in the spinal cords of *Mfn2^K357T/K357T^* compared to *Mfn2^+/+^* and *Mfn2^+/K357T^* mouse pups. *Mfn2^+/K357T^* mouse pups also exhibited mitochondrial clustering, albeit to a much lesser degree, without reaching significance compared to *Mfn2^+/+^* mice. Thus, altered mitochondrial dynamics in *Mfn2^K357T/K357T^* mouse pups seem to halt development and lead to postnatal lethality, and for this reason, only *Mfn2^+/K357T^* along with *Mfn2^+/+^* mice from both sexes were further utilized and analyzed at 6, 8, and 10 mos of age for the characterization of the *Mfn2^K357T^* KI mouse model.

### 2.3. Behavioral Performance and Sciatic Nerve Electrophysiological Properties of Mfn2^+/K357T^ Mice Are Not Affected up to 10 mo of Age

The weight (Figure S2A–C), motor coordination and balance (assessed by rotarod test) (Figure S2D–F), and muscle strength of four limbs (four-limb hang test) (Figure S2G–I) or hindlimbs (hindlimb grip strength test) (Figure S2J–L) of *Mfn2^+/K357T^* mice were not affected at 6, 8, and 10 mos of age. Moreover, their sciatic nerve electrophysiological properties showed no significant impairment compared to wild type littermates at 8 and 10 mos of age (Figure S3). Thus, *Mfn2^+/K357T^* mice remained asymptomatic up to 10 mos of age and showed no overall motor or electrophysiological abnormalities.

### 2.4. Histopathological Changes in Sciatic and Optic Nerves of Mfn2^+/K357T^ Mice

**Figure 2:**
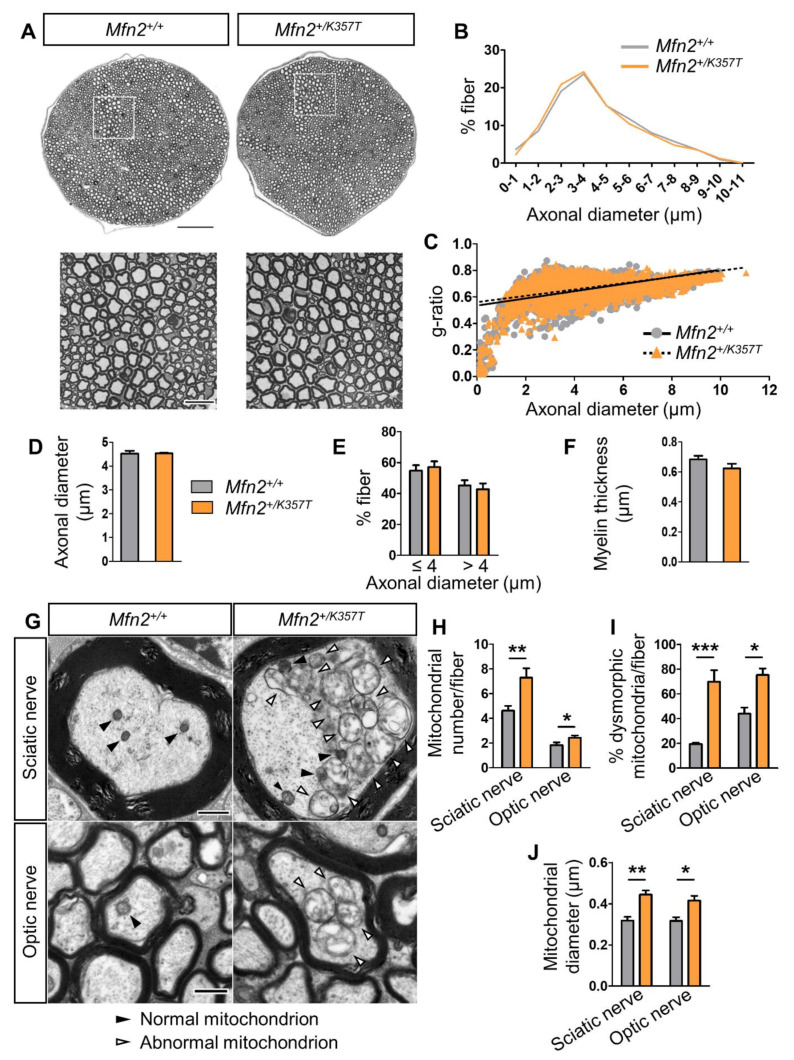
Morphometric analysis of sciatic nerves and ultrastructural analysis of axonal mitochondria in sciatic and optic nerves of *Mfn2^+/K357T^* mice. (**A**): Representative photos of sciatic nerve semithin cross sections from 10–mo–old *Mfn2^+/+^* and *Mfn2^+/K357T^* mice stained with toluidine blue, including overviews (scale bar = 100 μm) and magnified views (scale bar = 20 μm) from squared boxes. (**B**): Distribution of sciatic nerve fibers based on axonal diameter from *Mfn2^+/+^* and *Mfn2^+/K357T^* mice (*n* = ~500 fibers/mouse from *n* = 5 *Mfn2^+/+^* mice, and *n* = ~600 fibers/mouse from *n* = 5 *Mfn2^+/K357T^* mice). Two–way analysis of variance (ANOVA) with post hoc Bonferroni test was used. No statistical significance was reached. (**C**): G–ratio analysis scatterplots against axonal diameter (coefficient of determination: R^2^=0.2140 for *Mfn2^+/+^*, and R^2^=0.1925 for *Mfn2^+/K357T^*) from sciatic nerves of *Mfn2^+/+^* and *Mfn2^+/K357T^* mice. Unpaired Student’s *t*–test was used for average g–ratio comparison. No statistical significance was reached. (**D**–**F**): Comparison of average axonal diameter (**D**), percentages of small/medium (<4 μm) or large diameter axons (>4 μm) (**E**), and average myelin thickness (**F**) from all sciatic nerve fibers between *Mfn2^+/+^* and *Mfn2^+/K357T^* mice. Data are presented as mean ± SEM, and unpaired Student’s *t*–test was used for all comparisons. No statistical significance was reached. (**G**): Representative transmission electron microscopy photos of sciatic and optic nerve cross–sections from 8–mo–old *Mfn2^+/K357T^* and *Mfn2^+/+^* mice (scale bars = 500 nm). Black arrowheads indicate mitochondria with normal ultrastructure; white arrowheads indicate mitochondria with abnormal ultrastructure. (**H**–**J**): Counts of axonal mitochondria per fiber (**H**), percentage of mitochondria with an abnormal ultrastructure per fiber (**I**), and average diameter (**J**) of axonal mitochondria (from each mitochondrion the lesser diameter was considered) per fiber in sciatic and optic nerves of 8–mo–old *Mfn2^+/+^* and *Mfn2^+/K357T^* mice (*n* = 10 optic or sciatic nerve fibers/mouse from *n* = 3 mice/genotype). Data are presented as mean ± SEM. Two–way ANOVA with post hoc Bonferroni test was used for all comparisons: * *p* < 0.05, ** *p* < 0.01, *** *p* < 0.001.

Histological examination did not reveal any light-microscopic pathological changes in sciatic nerves from 10-mo-old *Mfn2^+/K357T^* mice compared to age-matched *Mfn2^+/+^* mice (Figure 2A). Morphometric analyses to quantify myelin and axon pathology in *Mfn2^+/K357T^* mice confirmed no changes between the two genotypes (Figure 2B–F). Segregation of fibers based on axonal diameter demonstrated a similar axon profile distribution between *Mfn2^+/+^* and *Mfn2^+/K357T^* sciatic nerves (Figure 2B), with no changes in their relative myelin sheath thickness assessed by g-ratios (Figure 2C). Average axonal diameter was identical between *Mfn2^+/+^* and *Mfn2^+/K357T^* mice (Figure 2D), as were the percentages of fibers with small/medium (<4 μm) and large (>4 μm) diameter axons (Figure 2E). Average myelin thickness was also similar between *Mfn2^+/+^* and *Mfn2^+/K357T^* mice (Figure 2F).

However, ultrastructural analysis of sciatic and optic nerve axons of 8-mo-old *Mfn2^+/K357T^* mice revealed an altered distribution of axonal mitochondria with aberrant clustering and abnormal morphology (Figure 2G). Due to mitochondrial clustering, a greater number of axonal mitochondria were detected in sciatic and optic nerve axons of *Mfn2^+/K357T^* than in *Mfn2^+/+^* mice (Figure 2H). Moreover, *Mfn2^+/K357T^* sciatic and optic nerves had a greater percentage of mitochondria with an altered morphology characterized by disorganized or absent cristae, delamination (detachment) of the outer and inner mitochondrial membranes (Figure 2I), and a pathological swelling leading to an increased mitochondrial diameter (Figure 2J). Thus, even though *Mfn2^+/K357T^* display altered mitochondrial dynamics with a dysmorphic mitochondrial profile, these changes do not result in functional impairment up to 10 mos of age.

### 2.5. Mitochondrial Clustering Increases with Age and Is More Pronounced in Sciatic and Optic Nerves of Mfn2^+/K357T^ Mice

**Figure 3:**
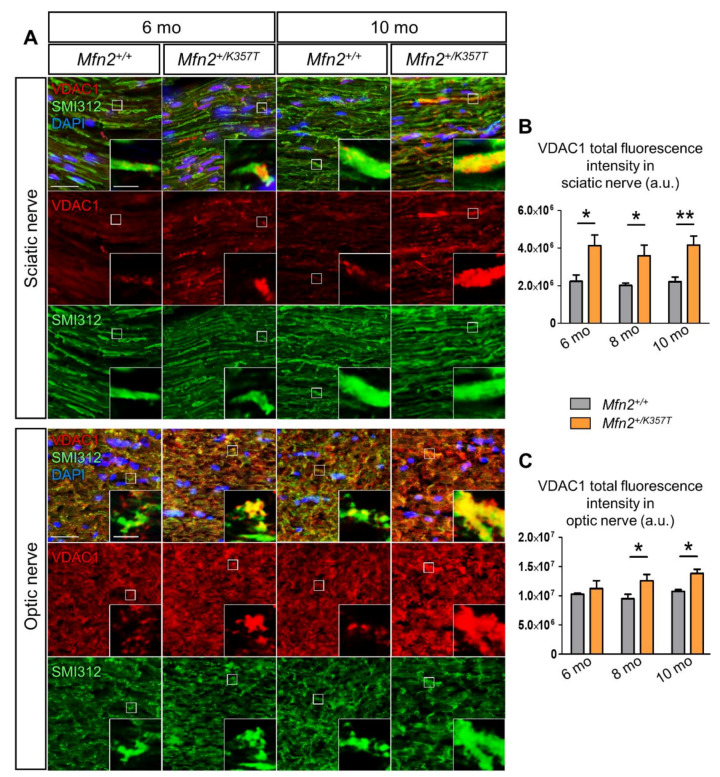

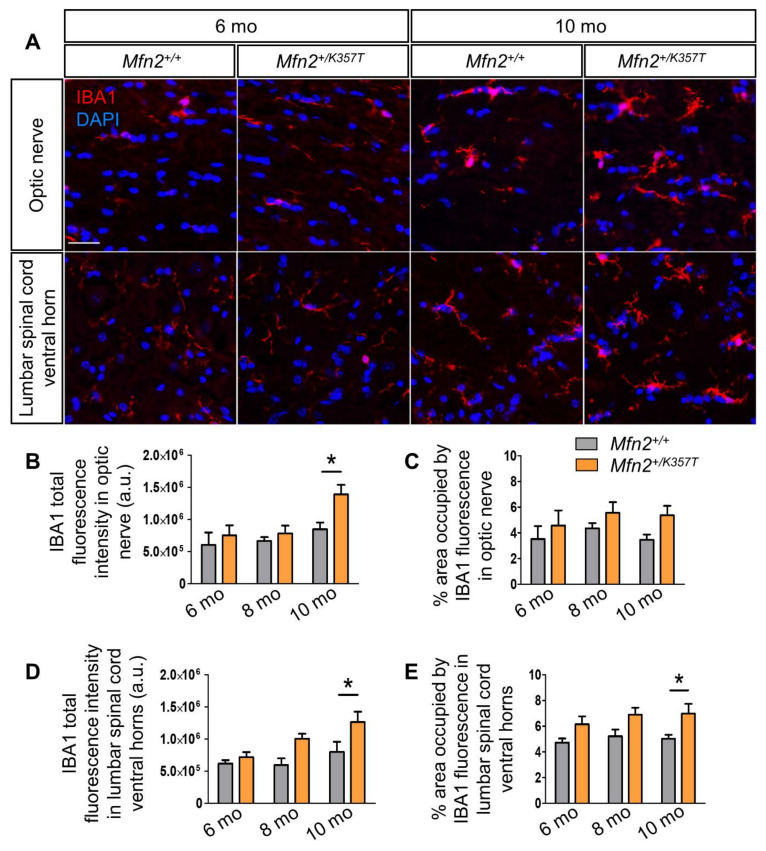
Evaluation of mitochondria localization in sciatic and optic nerves of *Mfn2^+/+^* and *Mfn2^+/K357T^* mice. (**A**): Representative immunofluorescence images of sciatic and optic nerve longitudinal sections as indicated from 6- and 10-mo-old *Mfn2^+/+^* and *Mfn2^+/K357T^* mice. Sections were stained against mitochondrial marker VDAC1 (red) and axonal marker SMI312 (green), and counterstained with DAPI (blue). Single and merged channels are shown. Magnified views (scale bar = 5 μm) from squared boxes in overviews (scale bar = 30 μm) are depicted in insets. Magnified views from *Mfn2^+/K357T^* mice portray sciatic and optic nerve axons occupied by VDAC1–positive mitochondrial clusters. (**B**,**C**): Semi–quantitative analysis of VDAC1 total fluorescence intensity in sciatic ((**B**); *n* = 5 mice/genotype) and optic nerve ((**C**); *n* = 4 mice/genotype for 6 mos, and *n* = 5 mice/genotype for 8 mos and 10 mos) longitudinal sections from *Mfn2^+/+^* and *Mfn2^+/K357T^* mice measured in 6 15 × 15 cm regions of interest (ROI) obtained from two 40 X images per mouse (3 ROI/image). a.u., arbitrary units. Data are presented as mean ± SEM. Two–way ANOVA with post hoc Bonferroni test was used for all comparisons; * *p* < 0.05, ** *p* < 0.01.

Sciatic and optic nerve tissues were stained with mitochondrial marker VDAC1 and with axonal neurofilament marker SMI312 to explore mitochondrial clustering in 6-, 8-, and 10-mo-old *Mfn2^+/K357T^* mice. VDAC1-immunoreactive mitochondrial clusters were progressively visualized inside sciatic and optic nerve axons (Figure 3A). Quantification in sciatic nerves confirmed increased VDAC1 total fluorescence intensity in *Mfn2^+/K357T^* mice in all age groups examined (Figure 3B), while VDAC1 total fluorescence intensity quantification in optic nerves revealed significant elevation at 8 and at 10 mos of age (Figure 3C).

Mitochondrial clustering was additionally studied in lumbar spinal cord motor neurons and in the adjacent white matter. Lumbar spinal cords from 6-, 8-, and 10-mo-old mice were stained for VDAC1 along with the neuronal marker NeuN or SMI312 for axons (Figure S4A). While some VDAC1 fluorescent clusters were spotted inside *Mfn2^+/K357T^* motor neuron cell bodies, clusters in white matter axons of *Mfn2^+/K357T^* mice became more pronounced at 10 mos (Figure S4A). Accordingly, VDAC1 mean fluorescence intensity in *Mfn2^+/K357T^* NeuN^+^ motor neuron cell bodies showed no significant elevation up to 10 mos of age (Figure S4B), while VDAC1 total fluorescence intensity in *Mfn2^+/K357T^* lumbar spinal cord white matter was significantly elevated only at 10 mos (Figure S4C). Taken together, sciatic and optic nerves seem more prone to mitochondrial clustering, likely because they carry longer axons, followed by white matter axons near spinal motor neurons.

### 2.6. Microgliosis but Not Astrogliosis in the CNS of 10-mo-old Mfn2^+/K357T^ Mice

**Figure 4:**
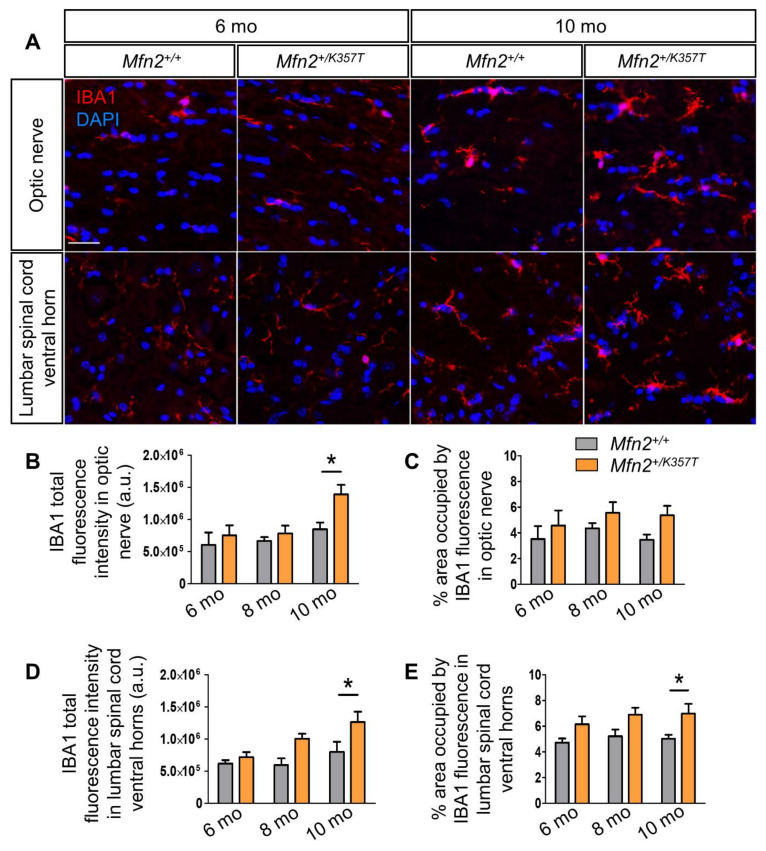
Microglia activation in optic nerves and lumbar spinal cords of *Mfn2^+/K357T^* mice. (**A**): Representative immunofluorescence images of optic nerve longitudinal sections and lumbar spinal cord ventral horn cross sections from 6- and 10-mo-old *Mfn2^+/+^* and *Mfn2^+/K357T^* mice. Sections were stained with microglia marker IBA1 (red) and counterstained with DAPI (blue) (scale bar = 30 μm). (**B**–**E**): Semi–quantitative analysis of IBA1 total fluorescence intensity (**B**,**D**) and area of occupancy (**C**,**E**) in optic nerve longitudinal sections from 6-mo-old (*n* = 4 mice/genotype), 8- and 10-mo-old (*n* = 5 mice/genotype) *Mfn2^+/+^* and *Mfn2^+/K357T^* mice (**B**,**C**) and in lumbar spinal cord ventral horns cross sections from 6-, 8-, and 10-mo-old *Mfn2^+/+^* and *Mfn2^+/K357T^* mice (*n* = 5 mice/genotype) (**D**,**E**), as indicated. IBA1 total fluorescence intensity and area were measured in 6 15 × 15cm ROI obtained from two 40× images per mouse (3 ROI/image). a.u., arbitrary units. Data are presented as mean ± SEM. Two–way ANOVA with post hoc Bonferroni test was used for all comparisons: * *p* < 0.05.

To evaluate whether *Mfn2^+/K357T^* mice develop inflammation in their CNS as reported in a previous CMT2A mouse model [29], 6-, 8-, and 10-mo-old optic nerves and lumbar spinal cords were stained for the microglial marker ionized calcium binding adaptor molecule 1 (IBA1) (Figure 4A), and the astrocytic marker glial fibrillary acidic protein (GFAP) (Figure S5A). Quantification of IBA1 total fluorescence intensity (Figure 4B) and total area (Figure 4C) in optic nerves showed no significant differences at 6 and 8 mos. However, at 10 mos, *Mfn2^+/K357T^* optic nerves presented with increased IBA1 total fluorescence intensity compared to *Mfn2^+/+^* mice, but showed no difference in IBA1 area. Similarly, quantification of IBA1 total fluorescence intensity (Figure 4D) and its total area (Figure 4E) in *Mfn2^+/K357T^* and *Mfn2^+/+^* lumbar spinal cord ventral horns displayed no difference at 6 mos or at 8 mos, but showed significant elevation in 10-mo-old *Mfn2^+/K357T^* mice.

To assess possible astrogliosis, GFAP total fluorescence intensity and area were measured in optic nerves and lumbar spinal cord ventral funiculi (Figure S5) of *Mfn2^+/+^* and *Mfn2^+/K357T^* mice at 6, 8, and 10 mos. No significant GFAP intensity and area alterations were detected in *Mfn2^+/K357T^* mice up to 10 mos of age. These findings show that *Mfn2^+/K357T^* mice are prone to neuroinflammation, and between CNS-residing microglia and astrocytes, microglia are the first to be mobilized.

### 2.7. Impaired Behavioral Performance and Increased Peripheral Inflammatory Response in Mfn2^+/K357T^ Mice Compared to Mfn2^+/+^ Mice at 4 h Post-LPS

Given previously published findings reporting a neuroprotective and anti-inflammatory role of *MFN2* [26], *Mfn2^+/K357T^* mice were additionally challenged with LPS in an effort to unmask CMT2A-relevant changes after exposure to a systemic inflammatory stimulus. Due to sex differences in immune responses after LPS-induced systemic inflammation—as male mice are more susceptible to sickness behavior [31,32], display a higher inflammatory response (i.e., IL-6, TNF-α) [32], and experience more severe LPS-induced tissue pathological changes [33]—only 6-mo-old male *Mfn2^+/+^* and *Mfn2^+/K357T^* mice were subjected to LPS treatment. A longitudinal study was conducted using the same mice groups for all LPS experiments, and mice were tested prior to LPS injection (0 h) and up to 96 h after LPS injection.

**Figure 5:**
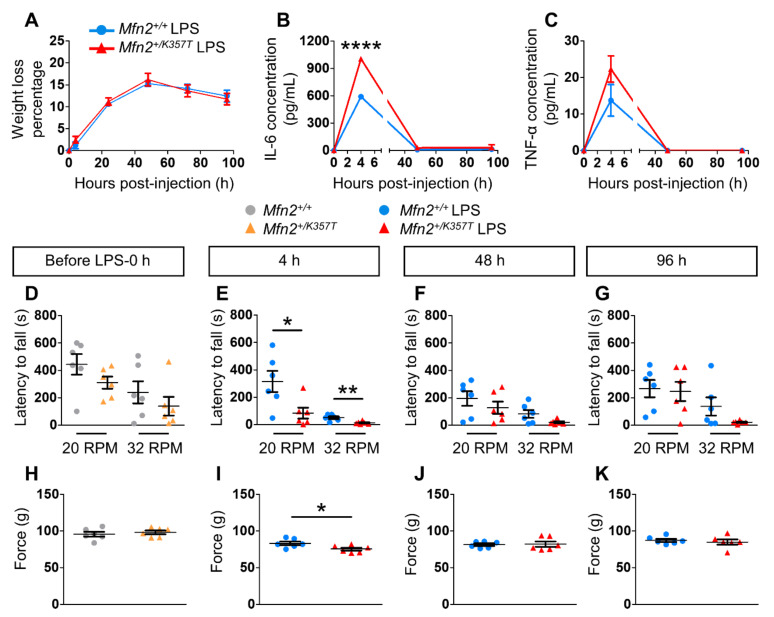
Inflammatory responses and behavioral changes in LPS–treated *Mfn2^+/+^* and *Mfn2^+/K357T^* mice. (**A**): Weight loss assessment of 6 mo *Mfn2^+/+^* and *Mfn2^+/K357T^* LPS–treated mice up to 96 h post–injection. (**B**,**C**): Assessment of IL–6 (**B**) and TNF–α (**C**) concentrations in serum samples collected at 0 h (before LPS) and at 4, 48, and 96 h post–LPS from *Mfn2^+/+^* and *Mfn2^+/K357T^* LPS–treated mice. Two–way ANOVA with post hoc Bonferroni test was used for all comparisons in (**A**–**C**): **** *p* < 0.0001. (**D**–**G**): Rotarod performance at 20 RPM and 32 RPM of 6-mo-old *Mfn2^+/+^* and *Mfn2^+/K357T^* LPS–treated mice at 0 h (no LPS, D) and at 4 h (**E**), 48 h (**F**), and 96 h (**G**) post–LPS. (**H**–**K**): Hindlimb grip strength of 6-mo-old *Mfn2^+/+^* and *Mfn2^+/K357T^* LPS–treated mice at 0 h (No LPS, H) and at 4 h (**I**), 48 h (**J**), and 96 h (**K**) post–LPS. The same 6-mo-old *Mfn2^+/+^* and *Mfn2^+/K357T^* mice groups were studied for all experiments (*n* = 6 mice/genotype). All data are presented as mean ± SEM. (**D**–**K**) Mann–Whitney U test was used for all comparisons: * *p* < 0.05, ** *p* < 0.01.

At 4 h after i.p. injection of a single sublethal dose of LPS (5 mg/Kg), all mice showed sickness behavior (lethargy, reduced mobility, loss of appetite, hunched posture, piloerection) and weight loss, followed by a gradual recovery. After 4 h and up to 48 h post-LPS, *Mfn2^+/+^* and *Mfn2^+/K357T^* mice experienced a transient weight loss, but started gradually gaining weight from 78 h until 96 h. The severity of sickness behavior and the degree of recovery did not differ between LPS-injected *Mfn2^+/+^* and *Mfn2^+/K357T^* mice, neither did their degree of weight loss at all time points tested (Figure 5A).

To examine the peripheral inflammatory response, IL-6 and TNF-α protein levels were assessed in serial serum samples from both genotypes before (0 h) and at 4 h, 48 h, and 96 h post-LPS injection. In both groups, IL-6 and TNF-α protein levels were below the detection limit at baseline conditions, but peaked at 4 h after LPS and then declined. IL-6 protein level was significantly higher in the *Mfn2^+/K357T^* LPS group than in the control *Mfn2^+/+^* LPS group at 4 h, but not at later time points of 48–96 h (Figure 5B). Because the detection limit for TNF-α was low and a higher volume of serum was required, sera samples were pooled into two samples containing sera from three individuals each. After LPS treatment, TNF-α protein levels were only detectable at 4 h where no statistically significant difference was observed between the two groups (Figure 5C).

At baseline conditions, *Mfn2^+/+^* and *Mfn2^+/K357T^* mice did not display significant differences in their rotarod performance at 20 and 32 rotations per minute (RPM) (Figure 5D), or in their hindlimb grip strength (Figure 5H). However, 4 h after LPS injection, *Mfn2^+/K357T^* LPS mice displayed a significant decrease in their rotarod performance at both speeds tested (Figure 5E), and a significant decrease in their hindlimb grip strength (Figure 5I). At 48 h (Figure 5F,J) and 96 h (Figure 5G,K) post-LPS, rotarod performance at both speeds tested (Figure 5F,G) and hindlimb grip strength (Figure 5J,K) did not differ significantly between the LPS-treated groups. Overall, *Mfn2^+/K357T^* mice showed an elevated peripheral peak response 4 h after LPS injection, accompanied by motor dysfunction compared to controls; however, they all recovered functionally at later time points similarly to wild type mice.

### 2.8. Pronounced Microgliosis in the CNS of Mfn2^+/K357T^ Mice Compared to Mfn2^+/+^ Mice at 96 h Post-LPS

**Figure 6:**
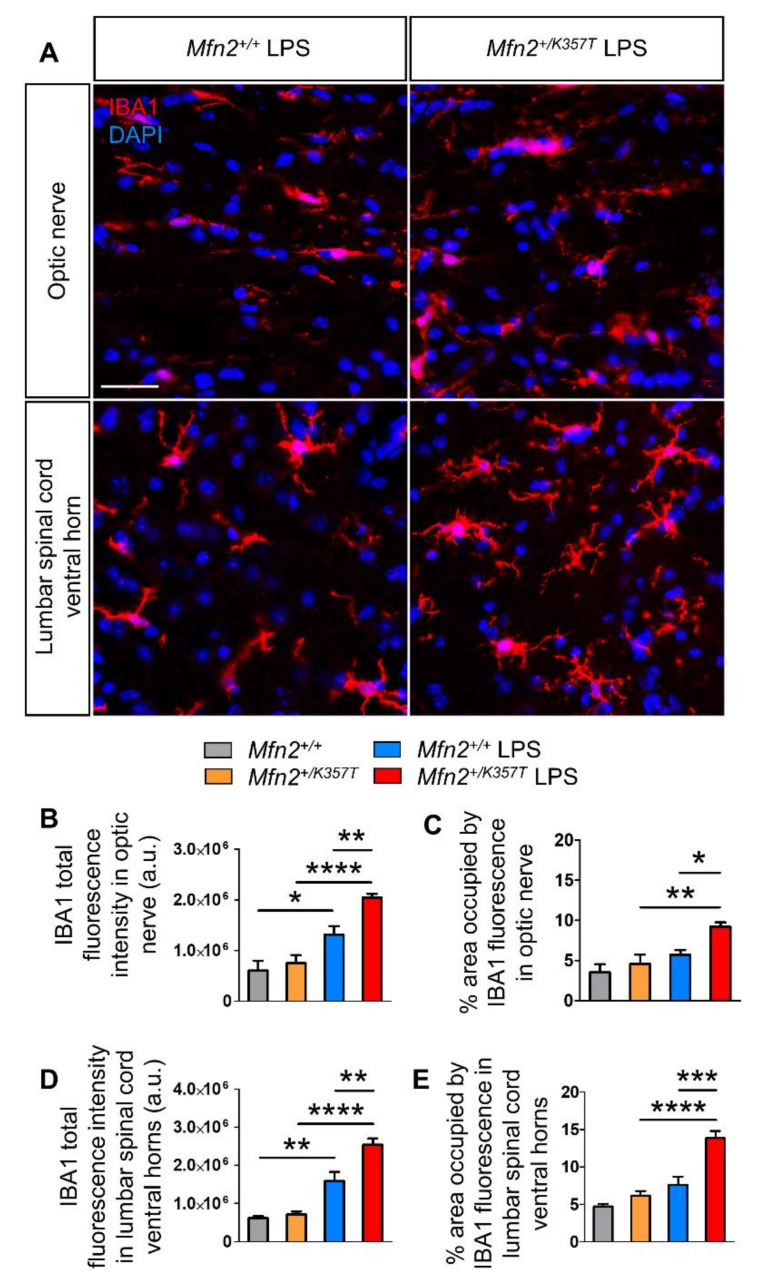
Exacerbated CNS inflammation in LPS–injected *Mfn2^+/K357T^* mice. (**A**): Representative immunofluorescence images of optic nerve longitudinal sections and lumbar spinal cord ventral horn cross sections from 6–mo–old *Mfn2^+/+^* and *Mfn2^+/K357T^* mice 96 h after LPS injection. Sections were stained against IBA1 for microglia (red) and counterstained with DAPI (blue) (scale bar = 30 μm). (**B**–**E**): Semi–quantitative analysis of IBA1 total fluorescence intensity (**B**,**D**) and area of occupancy (**C**,**E**) in optic nerve (**B**,**C**) and spinal cord (**D**,**E**) sections from 6–mo–old *Mfn2^+/+^* and *Mfn2^+/K357T^* mice at baseline conditions (without LPS injection; *n* = 4–5 mice/genotype), and 96 h after LPS injection (*n* = 6 mice/genotype). IBA1 total fluorescence intensity and area were measured in 6 15 × 15 cm ROI obtained from two 40× images per mouse (3 ROI/image). a.u., arbitrary units. Data are presented as mean ± SEM. One–way ANOVA with post hoc Tukey’s honestly significant difference (HSD) test was used for all comparisons: * *p* < 0.05, ** *p* < 0.01, *** *p* < 0.001, **** *p* < 0.0001.

LPS-mediated peripheral inflammation can induce neuroinflammation and activate astrocytes and microglia, cells of innate immunity in the CNS [26,34,35,36,37,38]. For this reason, the activation state of microglia (Figure 6A) and astrocytes (Figure S6) were assessed in *Mfn2^+/+^* and *Mfn2^+/K357T^* mice 96 h after LPS injection.

IBA1 total fluorescent intensity (Figure 6B) and area (Figure 6C) were assessed in optic nerves of 6-mo-old *Mfn2^+/+^* and *Mfn2^+/K357T^* LPS-treated mice, and were compared to baseline conditions in the absence of LPS. IBA1 intensity but not area in *Mfn2^+/+^* LPS, and IBA1 intensity and area in *Mfn2^+/K357T^* LPS mice were higher compared to *Mfn2^+/+^* and *Mfn2^+/K357T^* mice, respectively. Furthermore, IBA1 intensity and area were higher in *Mfn2^+/K357T^* LPS compared to *Mfn2^+/+^* LPS optic nerves. IBA1 total fluorescent intensity (Figure 6D) and area (Figure 6E) were also measured in lumbar spinal cord ventral horns of *Mfn2^+/+^* and *Mfn2^+/K357T^* LPS-treated mice and were compared to baseline conditions. IBA1 intensity but not area was higher in *Mfn2^+/+^* LPS than in *Mfn2^+/+^* mice, and IBA1 intensity and area were higher in *Mfn2^+/K357T^* LPS than in *Mfn2^+/K357T^* mice. Comparison of LPS-treated *Mfn2^+/+^* and *Mfn2^+/K357T^* lumbar spinal cords revealed higher IBA1 total intensity and greater area of IBA1 occupancy in *Mfn2^+/K357T^* LPS mice.

In assessing possible astrogliosis after LPS treatment, although GFAP total fluorescent intensity was elevated in the optic nerves and lumbar spinal cord ventral funiculi of both *Mfn2^+/+^* and *Mfn2^+/K357T^* mice 96 h after LPS injection compared to baseline, no significant GFAP alterations were detected between the LPS-treated mice groups (Figure S6). Thus, LPS-induced microglia and astrocyte activation was prominent in both *Mfn2^+/+^* and *Mfn2^+/K357T^* mice, but only LPS-induced microgliosis was significantly exacerbated in *Mfn2^+/K357T^* mice.

### 2.9. Increased Infiltration of Peripheral Leukocytes, Especially Macrophages, into the CNS of Mfn2^+/K357T^ Compared to Mfn2^+/+^ Mice 96 h Post-LPS

**Figure 7:**
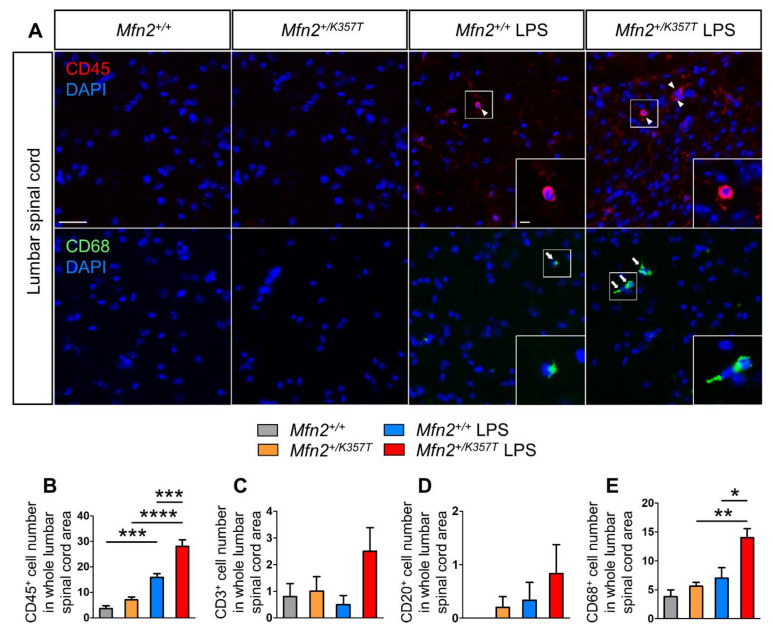
Assessment of CD45^+^, CD3^+^, CD20^+^, and CD68^+^ cell infiltrates in the lumbar spinal cord of LPS–treated *Mfn2^+/+^* and *Mfn2^+/K357T^* mice. (**A**): Representative immunofluorescence images of lumbar spinal cord cross sections from 6–mo–old *Mfn2^+/+^* and *Mfn2^+/K357T^* mice at baseline conditions (without LPS injection), and 96 h after LPS injection. Sections were stained against CD45^+^ for leukocytes (red; white arrowheads) and CD68^+^ for macrophages (green; white arrows), and counterstained with DAPI (blue). Magnified views (scale bar = 5 μm) from squared boxes in overviews (scale bar = 30 μm) are depicted in insets. (**B**–**E**): Counts of CD45^+^ (**B**), CD3^+^ (**C**), CD20^+^ (**D**), and CD68^+^ (**E**) cell infiltrates in whole lumbar spinal cord area of *Mfn2^+/+^* and *Mfn2^+/K357T^* mice at baseline conditions (*n* = 5 mice/genotype), and 96 h after LPS injection (*n* = 6 mice/genotype). Data are presented as mean ± SEM. One–way ANOVA with post hoc Tukey’s HSD test was used for all comparisons: * *p* < 0.05, ** *p* < 0.01, *** *p* < 0.001, **** *p* < 0.0001.

The extent of peripheral immune cell infiltration into the spinal cord parenchyma was also analyzed 96 h after LPS injection. Lumbar spinal cords were stained for CD3 (T-lymphocytes), CD20 (B-lymphocytes), CD45 (leukocyte common antigen, characterizes nucleated cells of hematopoietic origin), and CD68 (phagocytic cells) markers (Figure 7A). In the CNS, as CD45 additionally stains resting and activated microglia [39], and CD68 also stains activated phagocytic microglia [40], any CD45^+^ and CD68^+^ cells characterized by a microglial morphology were excluded from cell counting.

CD45 (Figure 7B) detects a range of leukocyte cell types, and hence, serves as a marker of overall cell infiltration in the CNS. During baseline conditions, CD45^+^ cell infiltrates did not reach statistical significance between *Mfn2^+/+^* and *Mfn2^+/K357T^* mice. After LPS treatment, CD45^+^ cells increased in *Mfn2^+/+^* LPS but significantly more in *Mfn2^+/K357T^* LPS mice, leading to more CD45^+^ infiltrates in the *Mfn2^+/K357T^* LPS than the *Mfn2^+/+^* LPS group. To check which leukocyte population infiltrated the CNS of *Mfn2^+/K357T^* LPS mice to a greater degree, the numbers of CD3^+^ (Figure 7C), CD20^+^ (Figure 7D), and CD68^+^ (Figure 7E) cell infiltrates were assessed. CD3^+^ cell counts were similar between *Mfn2^+/+^* and *Mfn2^+/K357T^* mice, and were not significantly altered after LPS injection. In *Mfn2^+/+^* mice, there was no detection of CD20^+^ cells, while in *Mfn2^+/K357T^* mice a negligible number was detected. After LPS injection, no significant difference was observed in CD20^+^ cell counts in either genotype compared to their baseline, nor did CD20^+^ cell counts differ significantly between the *Mfn2^+/K357T^* and *Mfn2^+/+^* LPS-treated groups. CD68^+^ cell infiltrates in *Mfn2^+/+^* and *Mfn2^+/K357T^* mice did not differ, but after LPS, a significant increase was detected in *Mfn2^+/K357T^* LPS but not in *Mfn2^+/+^* LPS mice, compared to baseline conditions. Direct comparison of LPS-injected *Mfn2^+/+^* and *Mfn2^+/K357T^* mice revealed a higher CD68^+^ cell number in the latter group. Taken together, LPS promoted minimal CNS infiltration in both groups 96 h after injection, but the *Mfn2^+/K357T^* LPS group was more prone to CNS infiltration of peripheral cells, especially macrophages.

### 2.10. No Significant Alterations in Mitochondrial Dynamics in Mfn2^+/+^ and Mfn2^+/K357T^ Mice 96 h after LPS Injection

Finally, to evaluate mitochondrial dynamics after LPS treatment, tissues from *Mfn2^+/+^* and *Mfn2^+/K357T^* LPS-injected mice were stained for VDAC1. Although LPS-induced inflammation may cause mitochondrial fragmentation [26,41], mitochondrial dynamics in each mouse group were not significantly altered 96 h post-LPS, compared to their respective baseline conditions (Figures S7 and S8). VDAC1 fluorescent clusters, significantly elevated only in *Mfn2^+/K357T^* sciatic nerves, persisted in LPS-treated *Mfn2^+/K357T^* sciatic nerves but did not reach statistical significance (Figure S7). Clusters were less evident in SMI312^+^ optic nerve axons (Figure S7), and NeuN^+^ motor neuron cell bodies and SMI312^+^ white matter axonal tracts of LPS-treated *Mfn2^+/K357T^* mice (Figure S8) and VDAC1 intensity did not differ compared to *Mfn2^+/+^* LPS mice. Thus, the exacerbated mitochondrial clustering in *Mfn2^+/K357T^* mice was not exacerbated 96 h after LPS-induced inflammation and their network was not significantly altered, indicating that they may be able to partly restore it by that time point.

## 3. Discussion

We generated a *Mfn2^K357T^*-expressing KI mouse model of CMT2A based on a novel *MFN2* mutation discovered in a CMT2A patient with early-onset severe neuropathy. This model showed subtle pathological and no functional changes at baseline, representing a pre-symptomatic CMT2A stage. While *Mfn2^+/K357T^* mice did not display disease signs, behavioral or electrophysiological abnormalities up to 10 mos of age, a more detailed examination revealed aberrant mitochondrial clustering, especially in their sciatic and optic nerves, with abnormal mitochondrial morphology and localization along with microglia activation in the CNS. Because of the mild phenotype, *Mfn2^+/K357T^* mice were further stressed by peripherally-induced neuroinflammation triggered by LPS injection. *Mfn2^+/K357T^* LPS mice developed a more severe peripheral inflammatory response and a significantly decreased performance 4 h after LPS injection, along with increased CNS infiltration and microglial activation 96 h after LPS compared to *Mfn2^+/+^* LPS mice, also highlighting the role of MFN2 function in immune responses.

Axonal degeneration and loss, and myelin defects were not observed in the sciatic nerves of *Mfn2^+/K357T^* mice, even up to 10 mos, which was further supported by their unaltered sciatic CMAP amplitudes and MNCVs. Therefore, motor coordination and fore- and hindlimb strength of *Mfn2^+/K357T^* mice remained unaffected. Contrastingly, *Mfn2^+/K357T^* mice displayed pronounced mitochondrial clustering in sciatic nerves starting from 6 mos, in optic nerves from 8 mos, in lumbar spinal cord white matter (occupied by axon tracts) at 10 mos, while no mitochondrial clustering was observed in lumbar motor neuron cell bodies up to 10 mos. The more pronounced mitochondrial clustering at an earlier timepoint in sciatic nerves marks their higher predisposition towards mitochondrial clustering and susceptibility to CMT2A disease, similarly to CMT2A patients whose peripheral nerves innervating their lower extremities are more affected [5,6,7,10,11]. Why peripheral nerves are more susceptible to the deleterious effects of *MFN2* mutations, which are ubiquitously expressed, is not yet clear. However, some mechanisms have been proposed, including the extreme axonal length of peripheral nerves that necessitate extensive mitochondrial transport which is interrupted by *Mfn2* mutations [21], and the lower *Mfn1* expression in peripheral nerves, which deprives them of adequate *Mfn1* that may complement mutant *Mfn2* [27].

In all tissues tested, mitochondrial clustering in *Mfn2^+/K357T^* mice appeared more pronounced with age, especially in sciatic nerves, due to a progressive build-up of mitochondrial clusters. The K357 of MFN2 is one of the critical sites necessary for the deacetylase sirtuin 1 (SIRT1)-mediated mitophagy (selective autophagy of damaged mitochondria) [42], and *Mfn2^K357T^* may affect this process. Mouse embryonic fibroblasts (MEFs) exclusively expressing *MFN2^R94Q^* displayed reduced mitophagy [30], and an increased number of neuronal mitochondrial clusters were found to be marked for mitophagy in *MFN2^R94Q^* transgenic mice, indicating an ineffective elimination of these clusters [29]. Moreover, mitophagy is a much slower process in neurons compared to non-neuronal cells [43,44], and this may be another factor rendering peripheral nerves more susceptible to *MFN2* mutations, as their greater axonal length seems to be a rate-limiting factor for mitophagy.

Ultrastructural analyses of axonal mitochondria in sciatic and optic nerves from 8-mo-old *Mfn2^+/K357T^* mice revealed mitochondrial aggregates. Mitochondria in these aggregates appeared attached to each other (tethered) via their outer membranes but were not fused, indicating a compromised mitochondrial fusion. The *Mfn2^K357T^* mutation appears to permit tethering but hinder fusion, giving rise to ‘sticky’ fusion-deficit mitochondria [22]. These ‘sticky’ mitochondria are trapped and accumulate in clusters, and because they cannot be efficiently eliminated or fuse efficiently with other mitochondria, they begin manifesting morphological traits characteristic of mitochondrial damage including disorganized or absent cristae, delamination of the outer and inner mitochondrial membrane, and swelling.

*Mfn2^K357T/K357T^* mouse pups displayed early postnatal lethality with a variation in their time of death (a greater mortality was observed at P0), presumably due to global abolishment of effective mitochondrial fusion and/or other *Mfn2*-mediated functions. All *Mfn2^K357T/K357T^* pups were phenotypically normal at birth, but *Mfn2^K357T/K357T^* pups surviving beyond P6 showed severe developmental delay, underlying the essential role of *Mfn2* in early postnatal development.

*Mfn2^+/+^* and *Mfn2^+/K357T^* mice subjected to systemic LPS injection at 6 mos experienced a deterioration in their motor coordination and hindlimb strength, with higher IL-6 and TNF-α serum levels as a peripheral immune response, which all peaked at 4 h and began to decline by 48 h post-LPS due to recovery. These alterations were more pronounced in *Mfn2^+/K357T^* compared to *Mfn2^+/+^* LPS-treated mice at 4 h post-LPS, except for TNF-α serum levels, probably due to the small number of serum samples tested for TNF-α, as samples had to be pooled. The *Mfn2^K357T^* mutation in this KI model is expressed in all cell types that physiologically express *Mfn2*. Therefore, the dysregulated mitochondrial fusion and/or other *Mfn2*-mediated functions in immune (peripheral blood cells [45,46]) and non-immune cells (muscle cells [47] and fibroblasts [48] to name a few) may have cumulatively contributed to the heightened peripheral IL-6 pool after exposure to LPS. Thus, our study provides further insights into the role of MFN2 in the regulation of the immune system; however, in order to clarify the contribution of this function to the CMT2A phenotype, further studies are required.

LPS induces both microglial and BBB endothelial activation, which facilitate CNS infiltration of peripheral leukocytes, including monocyte-derived macrophages [49,50]. Systemic inflammation by LPS triggered microgliosis, astrocytic activation, and CNS infiltration of peripheral leukocytes in *Mfn2^+/+^* and *Mfn2^+/K357T^* mice 96 h post-LPS, and these changes, apart from astrocytic activation, were more prominent in *Mfn2^+/K357T^* LPS-treated mice. At baseline conditions, *Mfn2^+/K357T^* mice did not experience astrocytic activation, but displayed microgliosis in their optic nerves and lumbar spinal cords at 10 mos. Baseline microgliosis could either result from the pronounced mitochondrial clustering in these tissues at 10 mos or directly from *Mfn2^K357T^* expression by microglia, or from both. In the presence of LPS, however, *Mfn2^+/K357T^* microglia became more reactive at 6 mos. This was probably due to a partly compromised mitochondrial fusion in these cells, as LPS-induced mitochondrial fragmentation in microglia, caused from excessive mitochondrial fission, augmented their expression of pro-inflammatory mediators (i.e., IL-6, TNF-α etc.) [41]. Furthermore, neuronal *MFN2* overexpression rescued LPS-induced mitochondrial fragmentation in neurons by promoting mitochondrial fusion, which subsequently suppressed microglial activation and IL-1β expression in the CNS, thus protecting mice from LPS-induced neuroinflammation [26]. Therefore, mitochondrial fusion appears to be essential for attenuation of inflammation and recovery after exposure to LPS, and cells with compromised mitochondrial fusion may be more susceptible to the detrimental effects of LPS.

While mitochondrial dynamics in CNS and PNS tissues did not differ significantly between *Mfn2^+/+^* and *Mfn2^+/K357T^* mice 96 h post-LPS, *Mfn2^+/K357T^* LPS-treated mice displayed a trend towards a more fragmented mitochondrial network. This non-significant LPS-induced fragmentation did not appear to resolve mitochondrial clusters as they persisted in *Mfn2^+/K357T^* LPS-treated mice, especially in their sciatic nerves, which are more prone to mitochondrial clustering as viewed from baseline conditions. This is why VDAC-1 fluorescence in 6-mo-old *Mfn2^+/K357T^* LPS sciatic nerves was less diminished compared to other *Mfn2^+/K357T^* LPS tissues tested. Taken together, ubiquitous *Mfn2^K357T^* expression levels, when in heterozygosity, did not exacerbate CMT2A-related disease manifestations, but rather affected peripheral inflammation, microglial activation, and CNS infiltration after LPS challenge.

The advantages of this model are that the *Mfn2^K357T^* mutation is in heterozygosity and its expression is driven by the *Mfn2* promoter, thus, more closely mimicking natural disease state in CMT2A patients who mostly harbor *MFN2* mutations in heterozygosity, and whose expression is dictated by the *MFN2* promoter [5,6,7,11,51,52]. However, mice were asymptomatic, as was the case with other KI mouse models that were either asymptomatic or developed late-onset CMT2A manifestations [28,53]. This discrepancy between CMT2A disease and related mouse models in regards to onset and severity could be attributed to biological and anatomical differences between the two species. The most successful attempts in modeling a severe, early-onset CMT2A in mice were with transgenic approaches [29,54,55] because their technology permits nerve tissue-specific heightened expression of *MFN2* mutations (depending on the promoter utilized), as CMT2A disease severity appears to be dose-dependent.

The limitations of this study are that spinal cord white matter and optic and sciatic nerves, apart from axons, contain other cell populations as well, which have been inevitably included during VDAC1 fluorescence intensity quantification. The low number of serum samples for TNF-alpha analysis may have diminished statistical significance. Furthermore, it would be useful to additionally evaluate LPS-induced histological changes in mice at peak response (4 h post-LPS) when behavioral and inflammatory changes reached their maximum. Further functional studies should also address the impact of the *Mfn2^K357T^* mutation on axonal mitochondrial transport, mitochondrial function (e.g., ATP generation, reactive oxygen species (ROS) production, regulation of mitochondrial membrane potential, mitochondrial dehydrogenase activity, cytochrome *c* release), and whether or not it has an influence on the proteins regulating mitochondrial fission.

## 4. Materials and Methods

### 4.1. Generation of CMT2A KI Mice Expressing Mfn2^K357T^

C57BL/6N mice were obtained from Koatech (Pyeongtaek, Korea). All mice were kept in controlled environmental conditions, maintained in individually ventilated cages, and given access to food and water *ad libitum*. This study was approved by the Institutional Animal Care and Use Committees of Seoul National University and Cyprus Institute of Neurology and Genetics, and was conducted in accordance with approved guidelines (license nr. CY/EXP/PR.L1/2017, date of approval: 19 June 2017, expiration date: 18 June 2022). For the purpose of this project, a CMT2A KI mouse model carrying the *Mfn2^K357T^* mutation was generated using the CRISPR/Cas9 genome editing and single-stranded oligodeoxynucleotide (ssODN)-mediated repair approach as described previously [56]. In brief, a solution containing Cas9 mRNA (50 ng/μL; ToolGen, Seoul, Korea), three overlapping single guide RNAs (sgRNAs; 10–20 ng/μL each) synthesized using an in vitro RNA synthesis kit (Thermo-Fisher Scientific, Waltham, MA, USA) following PCR amplification, and the ssODN (20 ng/μL) donor template (Integrated DNA Technologies, Coralville, IA, USA) with the *Mfn2^K357T^* mutation was microinjected into C57BL/6N zygotes. Overlapping sgRNAs (sharing at least 5 bps of the target *Mfn2* sequence site; Table 1) first introduced Cas9-mediated double-strand breaks (DSBs) at the mouse endogenous *Mfn2* locus. Then, in the presence of the ssODN donor template, DSBs underwent homology-directed repair (HDR) and the *Mfn2^K357T^*-encoding ssODN was finally integrated in the *Mfn2* locus. The use of overlapping sgRNAs has been previously shown to improve ssODN-mediated KI efficiency by favoring the HDR pathway [56].

Mice were genotyped with PCR amplification of crude tail lysates from tail-clipped juvenile mice or of toe lysates from toe-clipped mouse pups, using the KI-F (5′-CGAGAGGCAGTTTGAGGTAAGT-3′), KI-R (5′-TCATACATGTAGGCTCTCACCG-3′) primer pair (Eurofins Genomics LLC, Louisville, KY, USA). Due to the early postnatal mortality observed in homozygous mouse pups, toe-clipping was performed as a mean for simultaneous animal marking and genotyping to monitor the time of death of homozygous mouse pups. The PCR amplicons were purified with standard ethanol precipitation method, and sequenced using fluorescence-based cycle sequencing with BigDye^TM^ Terminator V3.1 Cycle Sequencing kit (Applied Biosystems, Waltham, MA, USA) and the KI-F primer, following the manufacturer’s protocol.

### 4.2. Behavioral Testing

*Rotarod test:* For the rotarod performance test, mice need to be preconditioned before the actual test [57]. For the preconditioning, *Mfn2^+/+^* and *Mfn2^+/K357T^* mice (6, 8, and 10 mos) underwent three trials with a maximum of 600 s each and 15 min rest period in between for 3 consecutive days. Each trial was performed on a rotarod apparatus (Ugo Basile, Gemonio, Italy) gradually accelerating from 4 to 40 RPM. On the test day, day 4, mice were tested at two different set speeds; 20 and 32 RPM. For each set speed, three trials with a maximum of 600 s each and a 15 min rest period in between was performed. The time elapsed from the moment that the mouse was placed on the rotarod until the moment it fell from the rod (latency to fall) was recorded. Each trial, both during preconditioning and on the test day, was terminated when the mouse fell from the rod or remained on the rod for 600 s.

*Four limb wire hang test:* A previously published method was followed with modifications [58]. *Mfn2^+/+^* and *Mfn2^+/K357T^* mice (6, 8, and 10 mos) were placed in the center of a metal wire mesh screen. The mesh was then inverted and suspended approximately 50 cm above a 15 × 15 × 15 cm transparent plexiglass container filled with bedding to avoid physical injuries from falling. The time elapsed from the moment the mesh was inverted until the moment the mouse fell from the mesh to the container (latency to fall) was recorded. For each mouse tested, three trials with a maximum of 10 min each and a 5 min rest period between trials were performed.

*Hindlimb grip strength test:* To measure the grip strength generated by the mouse hind limbs alone, the partly blind grasping grid provided by the manufacturer (Ugo Basile, Gemonio, Italy) was utilized, following a previously published protocol [57]. *Mfn2^+/+^* and *Mfn2^+/K357T^* mice (6, 8, and 10 mos) were suspended by their tail and were slowly lowered towards the grasping grid, which was connected to the grip strength meter. Mice were positioned on the grasping grid in such a way that their hind limbs only would grasp the grid, while it was ensured that their forelimbs would only come into contact with the blind part of the grid. Then, mice were gently pulled by their tail towards the investigator until their hindlimbs released the grid, taking care that their forelimbs did not grab the grid. Hindlimb grip strength values were recorded as grams (g). Three consecutive trials were performed per mouse.

### 4.3. Sciatic Nerve Motor Conduction Velocity Studies

Motor nerve conduction velocity (MNCV) was measured in vivo from bilateral sciatic nerves of *Mfn2^+/+^* and *Mfn2^+/K357T^* mice (8 and 10 mos), according to published methods [59]. MNCV was calculated following stimulation in anesthetized animals using two stimulation sites, one near the sciatic notch and one distally at the ankle via bipolar electrodes with supramaximal square-wave pulses (5 V) of 0.05 ms. The latencies of the compound muscle action potentials (CMAP) were recorded by a bipolar electrode inserted between digits 2 and 3 of the hind paw and measured from the stimulus artifact to the onset of the negative M-wave deflection. MNCV was calculated by dividing the distance between the stimulating and recording electrodes by the result of subtracting distal from proximal latency.

### 4.4. LPS-Induced Systemic Inflammation

LPS from *Escherichia coli* (*E. coli*) serotype O127:B8 was purchased from Sigma-Aldrich. Mice were weighed prior to LPS administration and a single sublethal dose of 5 mg/Kg (5 μL/g LPS stock concentration 1 mg/mL) was injected intraperitoneally (i.p.) in 6-mo-old *Mfn2^+/+^* and *Mfn2^+/K357T^* male mice for the induction of systemic inflammation. The behavior and weight of injected mice were monitored daily up to the day of sacrifice; 96 h (h).

### 4.5. Blood Collection and Sandwich ELISA Analysis of Serum Cytokines

Blood collection and processing were performed following a previously published protocol with minor modifications [60]. Serial blood samples of approximately 100 μL each were collected from the tail vein of LPS-injected 6-mo-old *Mfn2^+/+^* and *Mfn2^+/K357T^* mice at 4 different time-points: before LPS injection (0 h), and 4 h, 48 h, and 96 h after LPS injection. Mice were placed in a restraint tube and their tails were directly immersed in warm water (~45 °C) for 30 sec to increase blood flow. Their tails were swabbed with 70% alcohol and then wiped dry with a sterile gauze. A superficial incision at one-third of the tail length from the tail tip and above the lateral tail vein was performed with a razor blade, and blood was collected in an Eppendorf tube. To stop blood flow, gentle pressure was applied on the incision site. For consecutive sampling from the same tail, each incision was made distal to the previous incision site, towards the base of the tail.

To recover serum, blood samples were incubated at 37 °C for 20 min to promote coagulation and were stored overnight at 4 °C. Next, samples were centrifuged at 3000 RPM for 10 min and the resulting supernatant was stored in aliquots at −80 °C until analysis.

Quantification of interleukin-6 (IL-6) and tumor necrosis factor alpha (TNF-α) protein levels in the serum of LPS-treated mice was performed with the IL-6 mouse uncoated ELISA kit (Invitrogen, Waltham, MA, USA) and the TNF-α mouse uncoated ELISA kit (Invitrogen, Waltham, MA, USA), respectively, following the manufacturer’s protocol. For the detection of IL-6, a 1:20 dilution of each sample was used per well (5 μL of blood serum + 95 μL 1 X ELISA/ELISPOT diluent), while for TNF-α a 1:10 dilution was used per well (10 μL of blood serum + 90 μL 1 X ELISA/ELISPOT diluent). All samples were tested in duplicates. Due to insufficient specimen volume and the higher volume needed for the TNF-α analysis as the detection limit for TNF-α was low, the sera samples from the six individuals per group and per time-point were pooled into two samples, so that each pooled sample contained sera from three individuals.

### 4.6. Tissue Processing and Immunofluorescence Staining

Mice were deeply anesthetized and were transcardially perfused with 0.9% saline solution, followed by 4% paraformaldehyde (PFA) diluted in 0.1 M phosphate buffer (PB) pH 7.2. Spinal cord, sciatic nerves, and optic nerves from *Mfn2^+/+^* and *Mfn2^+/K357T^* mice (6, 8, and 10 mos), and from LPS-injected *Mfn2^+/+^* and *Mfn2^+/K357T^* mice (6 mos) were dissected and post-fixed in 4% PFA for 1 h (spinal cord) or 30 min (sciatic and optic nerves). Mouse pups up to 6 days of age (postnatal day 6, P6) were deeply anesthetized, except for homozygous pups (*Mfn2^K357T/K357T^*) that were collected after death due to early postnatal lethality. Pups had their entire skin removed, their thoracic, peritoneal, and pelvic cavities were emptied of any visceral organs and were immersion fixed in 4% PFA for 24 h. Prior to freezing, tissues were incubated in 20% sucrose diluted in 0.1 M PB pH 7.2 to prevent the formation of ice crystals in the specimens. Tissues were then embedded and frozen in Tissue-Plus^®^ optimal cutting temperature (O.C.T.) compound (Scigen Scientific Inc., Gardena, CA, USA) and stored at −80 °C. Serial sections (12 μm thick, or 25 μm thick for mouse pups) were cut in a cryostat microtome and mounted on slides.

For the immunofluorescence (IF) staining, tissues were permeabilized with cold acetone for 10 min at −20 °C, and then blocked with 0.5% Triton X-100 in 5% BSA for 1 h at room temperature. Sections were then incubated at 4 °C overnight with the desired primary antibody in blocking solution: mouse anti-NeuN (Merck Millipore, Burlington, MA, USA, 1:400), mouse anti-SMI312 (BioLegend, San Diego, CA, USA, 1:100), mouse anti-GFAP (Invitrogen, Waltham, MA, USA, 1:300), rabbit anti-IBA-1 (Biocare Medical, Pacheco, CA, USA, 1:500), rabbit anti-VDAC1 (Abcam, Cambridge, UK, 1:200), rat anti-CD68:Alexa Fluor 488 (Serotec, 1:50), rabbit anti-CD3 (Abcam, Cambridge, UK, 1:100), goat anti-CD20 (Santa Cruz Biotechnology, Dallas, TX, USA, 1:100), and rat anti-CD45 (Abcam, Cambridge, UK, 1:100). The next day, sections were washed with 1X PBS, followed by a 1 h incubation at room temperature with the respective fluorescent dye coupled secondary antibody in blocking solution: anti-mouse FITC (Jackson ImmunoResearch, West Grove, PA, USA, 1:2000), anti-rabbit TRITC (Jackson ImmunoResearch, 1:3000), anti-goat FITC (Abcam, Cambridge, UK, 1:700), and anti-rat Alexa Fluor^®^ 555 (Invitrogen, Waltham, MA, USA, 1:2000). For the visualization of the cell nuclei, sections were counterstained with DAPI (Sigma-Aldrich, St. Louis, MO, USA), and coverslipped with fluorescence mounting medium (Dako Omnis, Agilent Technologies Inc., Santa Clara, CA, USA). Images were taken with a Nikon Eclipse Ni-E microscope with the DS-Qi2 camera head at 40× magnification using the NIS-Elements imaging software. Due to the thickness of mouse pups tissue sections, z-stack images at 40× magnification were acquired instead.

Quantification of IF images was carried out with ImageJ (Version 1.53c, Wayne Rasband, National Institutes of Health, Bethesda, MD, USA, http://imagej.nih.gov/ij). VDAC1 total fluorescence intensity (lumbar spinal cord white matter, optic and sciatic nerves of mature adult and middle-aged mice, and spinal cords of mouse pups), IBA1 total fluorescence intensity and area (lumbar spinal cord ventral horns), and GFAP total fluorescence intensity and area (lumbar spinal cord ventral funiculi) were measured from a total of six 15 × 15 cm regions of interest (ROI) from two images (three 15 × 15 cm ROI/image) per mouse. VDAC1 mean fluorescence intensity in motor neuron cell bodies was calculated from a total of *n* = 10 NeuN^+^ cell bodies from both lumbar spinal cord ventral horns (*n* = 5 NeuN^+^ cell bodies/ventral horn) per mouse. Prior to quantification, images were thresholded to exclude background fluorescence.

### 4.7. Tissue Processing and Morphometric Analyses of Sciatic Nerves

Mice were deeply anesthetized and were transcardially perfused with 2.5% glutaraldehyde in 0.1 M PB pH 7.2. Dissected sciatic nerves from 10 mo *Mfn2^+/+^* and *Mfn2^+/K357T^* mice were post-fixed in 2.5% glutaraldehyde at 4 °C for 24 h, and were processed for electron microscopy (EM) analysis.

Resin blocks were serially cross-sectioned, and semithin sections (1 μm) were stained with alkaline toluidine blue and imaged under a Nikon Eclipse Ni-E microscope with the DS-Fi2 camera head at 10× and 60× magnifications using the NIS-Elements imaging software. Images of sciatic nerve transverse sections obtained at a 60× magnification were analyzed with ImagePro-Plus (v6.0.0.260, by. Media Cybernetics, Inc., Rockville, MD, USA) to measure the axonal diameter, myelin thickness, and calculate the g-ratio of sciatic nerve fibers (~500–600 fibers per mouse).

### 4.8. Transmission Electron Microscopy Study of Sciatic and Optic Nerve Mitochondria

Sciatic and optic nerve resin blocks from 8-mo-old *Mfn2^+/+^* and *Mfn2^+/K357T^* mice were prepared as described above, and ultra-thin sections (0.1 μm) were obtained. Ultra-thin sections were stained with uranyl acetate and lead citrate and were imaged with a transmission electron microscope (TEM) at 4000×, 20,000×, and 30,000× magnification.

Mitochondria from sciatic and optic nerve fibers (10 fibers per mouse) were analyzed for ultrastructural and topological abnormalities by using TEM at 20,000× magnification. For the assessment of ultrastructural abnormalities, mitochondria were classified based on their appearance as either normal or dysmorphic (decreased electron density of the matrix, altered cristae morphology, mitochondrial swelling), and the diameter of all mitochondria (both normal and dysmorphic) per fiber was measured with ImageJ (Version 1.53c, Wayne Rasband, National Institutes of Health, Bethesda, MD, USA, http://imagej.nih.gov/ij). To assess topological abnormalities, the total number of mitochondria (both normal and dysmorphic) per fiber was counted.

### 4.9. Statistical Analyses

GraphPad Prism software (Version 5.01, GraphPad Software, San Diego, CA, USA, www.graphpad.com) was used for all data analyses and presentation. The statistical method used for each occasion is mentioned in figure legends. All data are presented as mean ± standard error of the mean (SEM), and statistically significant was considered any comparison with *p* < 0.05. For the comparison of two independent groups, unpaired two-tailed Student’s *t*-test was used or Mann–Whitney U-test for non-normally distributed data. For the comparison of three or more independent groups, regular (no pairing) one-way ANOVA with post-hoc Tukey’s HSD test, or regular (no pairing) two-way ANOVA with post hoc Bonferroni test were used, depending on the number of independent variables tested. For survival curve comparison the log-rank (Mantel–Cox) test was used.

## 5. Conclusions

In summary, we reported a novel CMT2A model with subtle disease manifestations. *Mfn2^K357T/K357T^* mice displayed a severe phenotype with early postnatal lethality, while *Mfn2^+/K357T^* mice manifested mild manifestations of CMT2A disease, including abnormal mitochondrial clustering, especially in their sciatic nerves, and delayed microgliosis. Injection of LPS in *Mfn2^+/K357T^* mice augmented their peripheral and CNS inflammation, increased CNS infiltration, and brought a transient reduction in their behavioral performance. These indicate that although heterozygous expression of the *Mfn2^K357T^* mutation altered mitochondrial dynamics and possibly other *Mfn2*-mediated functions, it was not sufficient to trigger a full CMT2A phenotype, even after LPS administration. To do so, a higher neuronal *Mfn2^K357T^* expression level may be required, as CMT2A disease is dose-dependent.

## Figures and Tables

**Table 1:** sgRNA sequences used in this study.

	sgRNA-PAM
sgRNA #1	ATTTGAGCAGCACACAGTCC-GGG
sgRNA #2	AATTTGAGCAGCACACAGTC-CGG
sgRNA #3	GACTGTGTGCTGCTCAAATT-TGG

## Data Availability

The data presented in this study are available within the article or supplementary material. All complete mRNA coding sequences and CDS sequences of human *MFN2* and mouse *Mfn2* reported in this study were collected from the publicly available National Center for Biotechnology Information (NCBI) Reference Sequence (RefSeq) Database (https://www.ncbi.nlm.nih.gov/refseq/) and the Consensus Coding Sequence (CCDS) Database (https://www.ncbi.nlm.nih.gov/CCDS/CcdsBrowse.cgi). The data used from these databases can be accessed by their respective RefSeq accession number or CCDS ID reported in the main text.

## Supplementary Materials

Page: 20The following are available online at https://www.mdpi.com/article/10.3390/ijms222111569/s1, Figure S1: Development of *Mfn2^K357T/K357T^* KI mice and characterization of spinal cord mitochondrial aggregation, Figure S2: Behavioural assessment of *Mfn2^+/K357T^* mice at 6, 8, and 10 mos of age, Figure S3: Electrophysiological assessment of 8 and 10–mo–old *Mfn2^+/K357T^* mice, Figure S4: Evaluation of mitochondrial localization in lumbar spinal cord motor neuron cell bodies and white matter of *Mfn2^+/+^* and *Mfn2^+/K357T^* mice, Figure S5: Lack of significant astrogliosis in optic nerves and lumbar spinal cords of *Mfn2^+/K357T^* mice, Figure S6: Astrogliosis in optic nerves and lumbar spinal cords of LPS–injected *Mfn2^+/+^* and *Mfn2^+/K357T^* mice, Figure S7: Mitochondrial localization in LPS–injected *Mfn2^+/+^* and *Mfn2^+/K357T^* mice, Figure S8: Mitochondrial localization in motor neuron cell bodies and white matter of LPS–injected *Mfn2^+/+^* and *Mfn2^+/K357T^* mice.
