# Supplementary material for: Aberrant Mitochondrial Dynamics and Exacerbated Response to Neuroinflammation in a Novel Mouse Model of CMT2A
Source: Int J Mol Sci. 2021 Oct 26;22(21):11569. doi: 10.3390/ijms222111569 (PMC8584238; doi:10.3390/ijms222111569)
Supplement: Supplementary file 1 [file ijms-22-11569-s001.zip › ijms-1399397-supplementary.pdf]

Aberrant Mitochondrial Dynamics and Exacerbated Response to Neuroinflammation in a Novel Mouse Model of CMT2A

Filippos Stavropoulos, Irene Sargiannidou, Louiza Potamiti, Alexia Kagiava, Mihalis I. Panayiotidis, Ji Hyun Bae, Su Cheong Yeom, Jae Young Lee and Kleopas A. Kleopa

Supplementary Materials

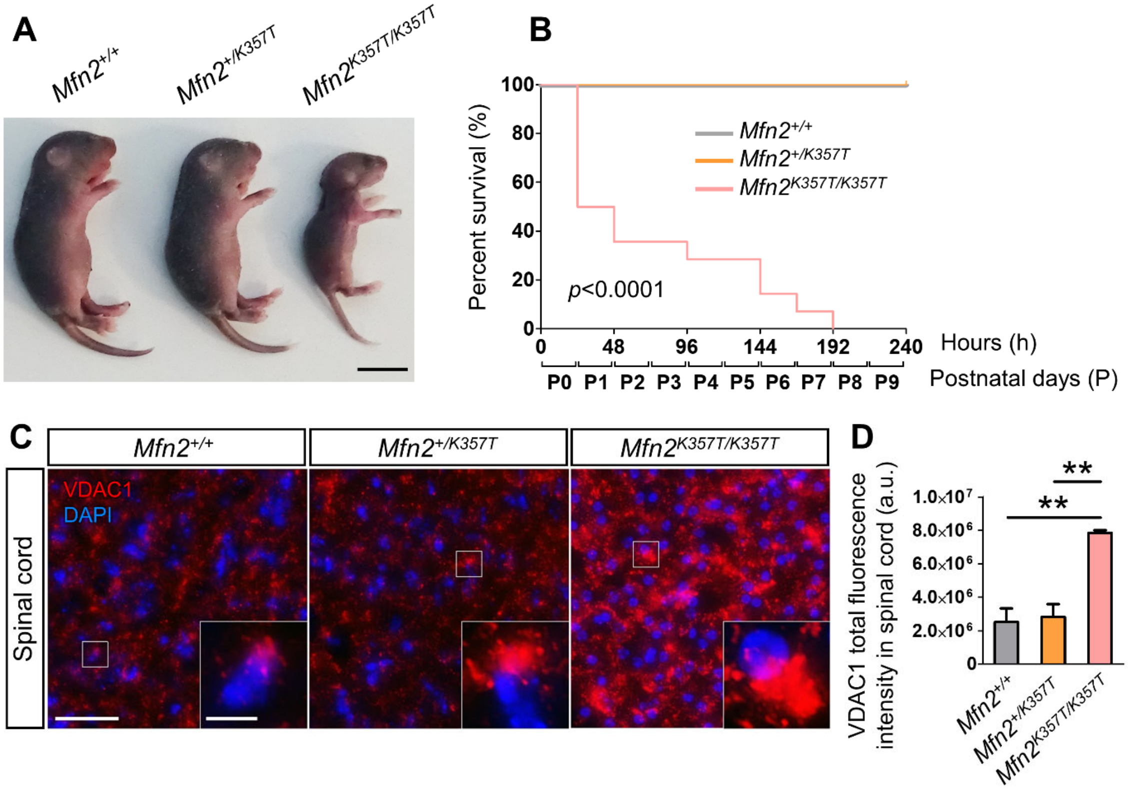

Figure S1. Development of *Mfn2*$^{K357T/K357T}$ KI mice and characterization of spinal cord mitochondrial aggregation. (**A**): Postnatal day 6 (P6) *Mfn2*$^{+/+}$, *Mfn2*$^{+/K357T}$, and *Mfn2*$^{K357T/K357T}$ mouse pups (scale bar = 1 cm). (**B**): Kaplan–Meier survival curves of *Mfn2*$^{+/+}$, *Mfn2*$^{+/K357T}$, and *Mfn2*$^{K357T/K357T}$ mouse pups (*n* = 14 pups/genotype) up to P9 (216–240 h). Survival curves were compared with the log–rank (Mantel–Cox) test. (**C**): Representative immunofluorescence images of spinal cord longitudinal sections from P6 *Mfn2*$^{+/+}$, P6 *Mfn2*$^{+/K357T}$, and P1 *Mfn2*$^{K357T/K357T}$ mouse pups. Sections were stained against mitochondrial marker VDAC1 (red), and counterstained with DAPI (blue). Magnified views (scale bar = 30 μm) from squared boxes in overviews (scale bar = 5 μm) are depicted in insets. *Mfn2*$^{+/K357T}$ and *Mfn2*$^{K357T/K357T}$ mice show cells occupied by VDAC1^{+} mitochondrial clusters, which are more prominent in *Mfn2*$^{K357T/K357T}$ mouse pups. (**D**): Semi–quantitative analysis of VDAC1 total fluorescence intensity in spinal cord longitudinal sections of *Mfn2*$^{+/+}$ (all P6), *Mfn2*$^{+/K357T}$ (all P6), and *Mfn2*$^{K357T/K357T}$ (P1, P4, P8) mouse pups (*n* = 3 mouse pups/genotype). VDAC1 total fluorescence intensity was measured in six 15 x 15 cm regions of interest (ROI) obtained from two 40 X Z–stack images per mouse pup (3 ROI/image). a.u., arbitrary units. Data are presented as mean ± SEM. One–way ANOVA with post hoc Tukey's HSD test: **p<0.01.

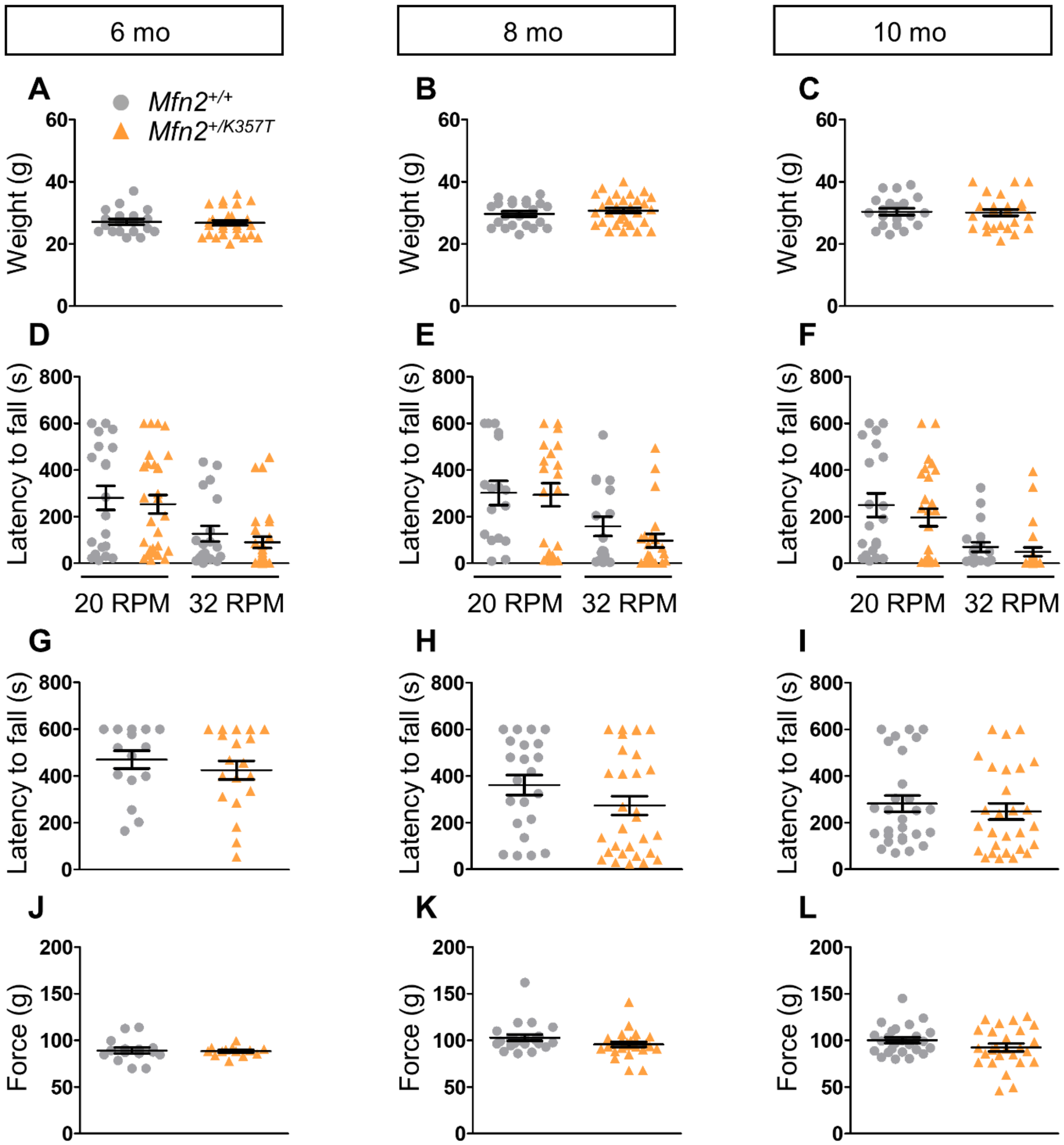

Figure S2. Behavioural assessment of *Mfn2*$^{+/K357T}$ mice at 6, 8, and 10 mos of age. (**A**–**C**): Weight assessment of 6–mo–old n = 19 *Mfn2*$^{+/+}$ and n = 29 *Mfn2*$^{+/K357T}$ mice (**A**), 8–mo–old n = 22 *Mfn2*$^{+/+}$ and n = 30 *Mfn2*$^{+/K357T}$ mice (**B**), and 10–mo–old n = 20 *Mfn2*$^{+/+}$ and n = 27 *Mfn2*$^{+/K357T}$ mice (**C**). Data are presented as mean ± SEM. Unpaired student's *t*–test was used for weight comparisons. No statistical significance was reached. (**D**–**F**): Rotarod performance at 20 RPM and 32 RPM of 6–mo–old n = 20 *Mfn2*$^{+/+}$ and n = 29 *Mfn2*$^{+/K357T}$ mice (**D**), 8–mo–old n = 17 *Mfn2*$^{+/+}$ and n = 22 *Mfn2*$^{+/K357T}$ mice (**E**), and 10–mo–old n = 20 *Mfn2*$^{+/+}$ and n = 27 *Mfn2*$^{+/K357T}$ mice (**F**). (**G**–**I**): Four limb wire hang test performance of 6–mo–old n = 16 *Mfn2*$^{+/+}$ and n = 19 *Mfn2*$^{+/K357T}$ mice (**G**), 8–mo–old n = 22 *Mfn2*$^{+/+}$ and n = 30 *Mfn2*$^{+/K357T}$ mice (**H**), and 10–mo–old n = 28 *Mfn2*$^{+/+}$ and *Mfn2*$^{+/K357T}$ mice (**I**). (**J**–**L**): Hindlimb grip strength of 6–mo–old n = 15 *Mfn2*$^{+/+}$ and n = 13 *Mfn2*$^{+/K357T}$ mice (**J**), 8–mo–old n = 22 *Mfn2*$^{+/+}$ and n = 26 *Mfn2*$^{+/K357T}$ mice (**K**), and 10–mo–old n = 25 *Mfn2*$^{+/+}$ and n = 26 *Mfn2*$^{+/K357T}$ mice (**L**). Data are presented as mean ± SEM. Mann–Whitney U test was used for all comparisons. No statistical significance was reached.

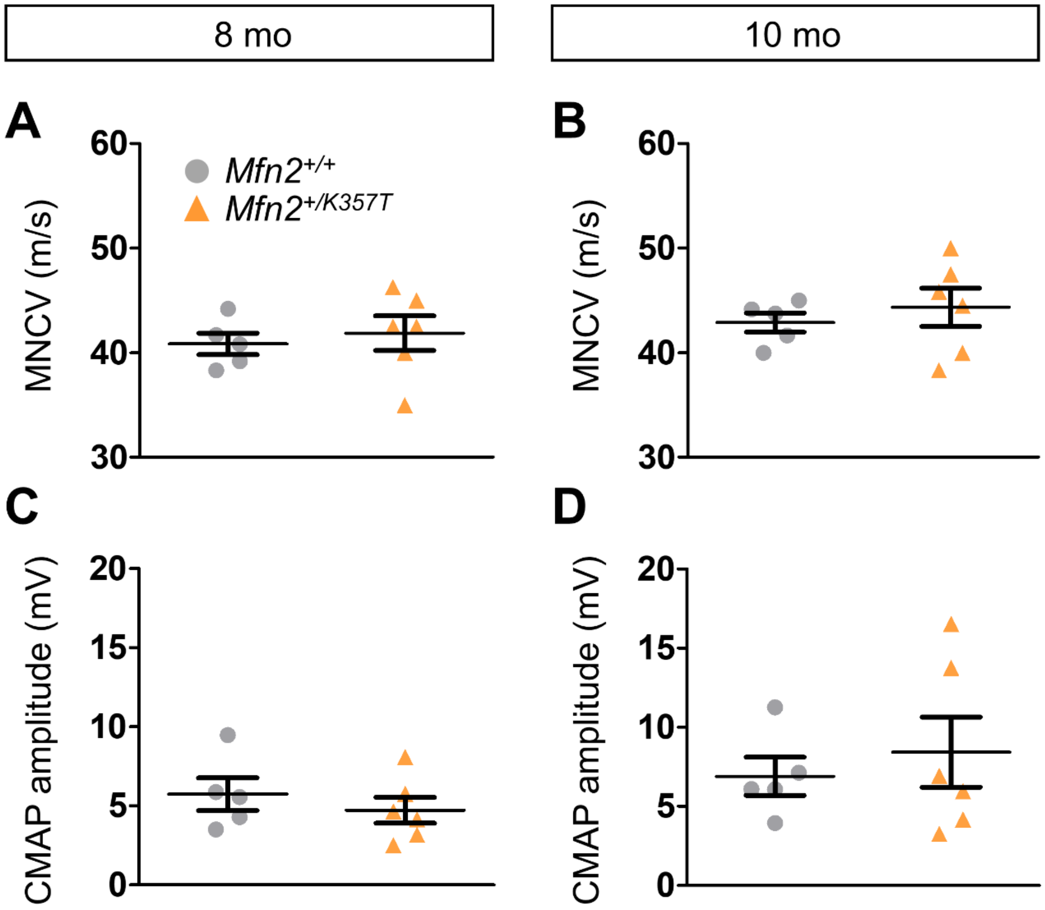

Figure S3. Electrophysiological assessment of 8 and 10–mo–old *Mfn2*$^{+/K357T}$ mice. Sciatic motor nerve conduction velocity (MNCV) in 8– (**A**) and 10–mo–old (**B**) *n* = 5 *Mfn2*$^{+/+}$ and *n* = 6 *Mfn2*$^{+/K357T}$ mice, and amplitude of the sciatic compound muscle action potential (CMAP) in 8– (**C**) and 10–mo–old (**D**) *n* = 5 *Mfn2*$^{+/+}$ and *n* = 6 *Mfn2*$^{+/K357T}$ mice. Data are presented as mean ± SEM. Mann–Whitney U test was used for all comparisons. No statistical significance was reached.

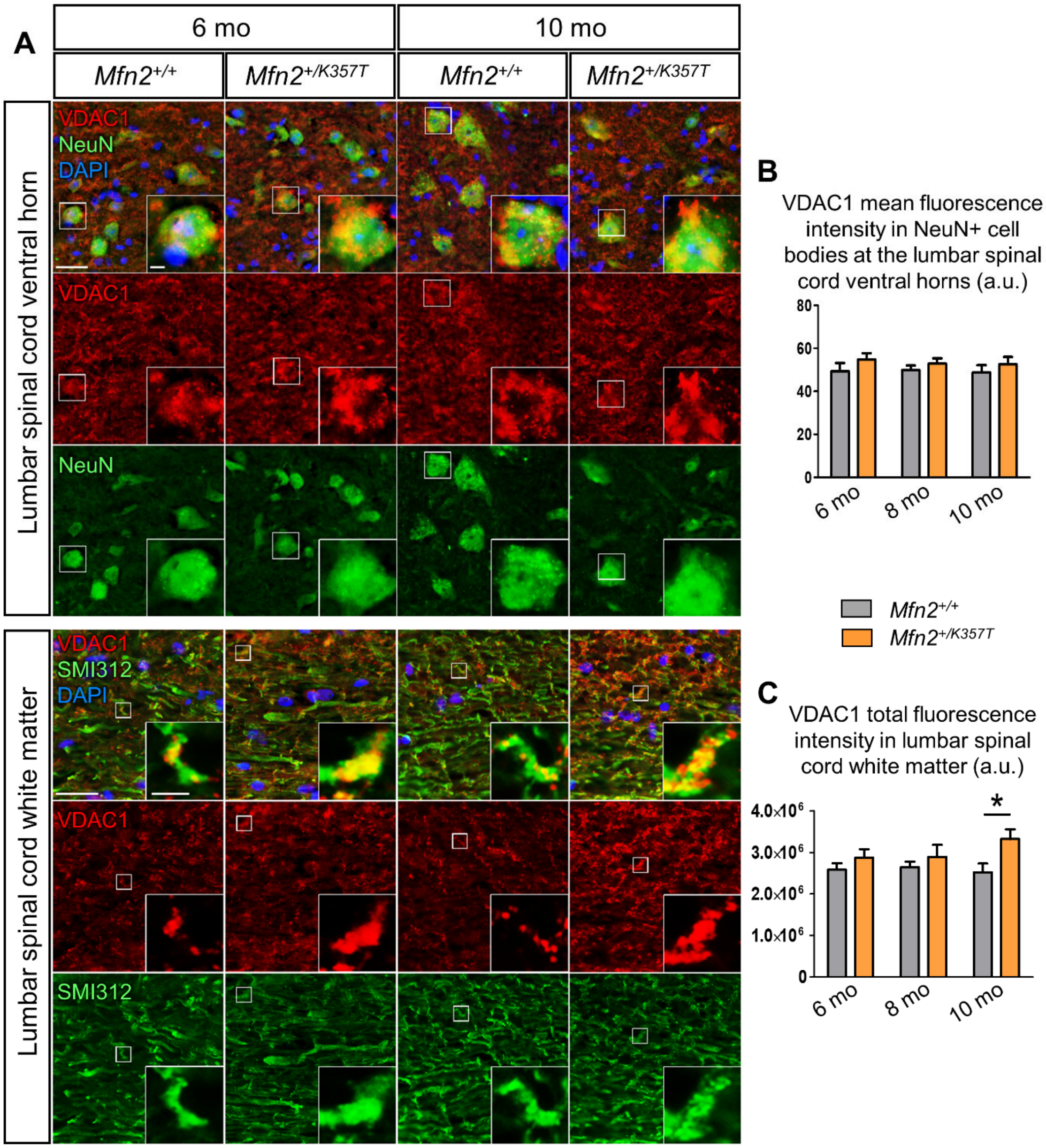

Figure S4. Evaluation of mitochondrial localization in lumbar spinal cord motor neuron cell bodies and white matter of *Mfn2*$^{+/+}$ and *Mfn2*$^{+/K357T}$ mice. (**A**): Representative immunofluorescence images of lumbar spinal cord motor neuron cell bodies in cross sections, and white matter longitudinal sections from 6– and 10–mo–old *Mfn2*$^{+/+}$ and *Mfn2*$^{+/K357T}$ mice. Sections were stained against VDAC1 for mitochondria (red), NeuN for neuronal cell bodies (green), SMI312 for axonal tracts (green), and counterstained with DAPI (blue). Single and merged channels are shown. Magnified views (scale bar = 5 μm) from squared boxes in overviews (scale bar = 30 μm) are depicted in insets. Magnified views from *Mfn2*$^{+/K357T}$ mice portray motor neuron cell bodies and white matter axons occupied by VDAC1^{+} mitochondrial clusters. (**B**): Semi–quantitative analysis of VDAC1 mean fluorescence intensity in motor neuron cell bodies from $n = 10$ NeuN–positive cell bodies/mouse. (**C**): Semi–quantitative analysis of VDAC1 total fluorescence intensity in the white matter measured in 6 15 x 15 cm regions of interest (ROI) obtained from two 40 X images per mouse (3 ROI/image). Quantifications were performed on 6–, 8–, and 10–mo–old *Mfn2*$^{+/+}$ and *Mfn2*$^{+/K357T}$ mice ($n = 5$ mice/genotype). a.u., arbitrary units. Data are presented as mean ± SEM. Two–way ANOVA with post hoc Bonferroni test was used for all comparisons; *$p<0.05$, **$p<0.01$.

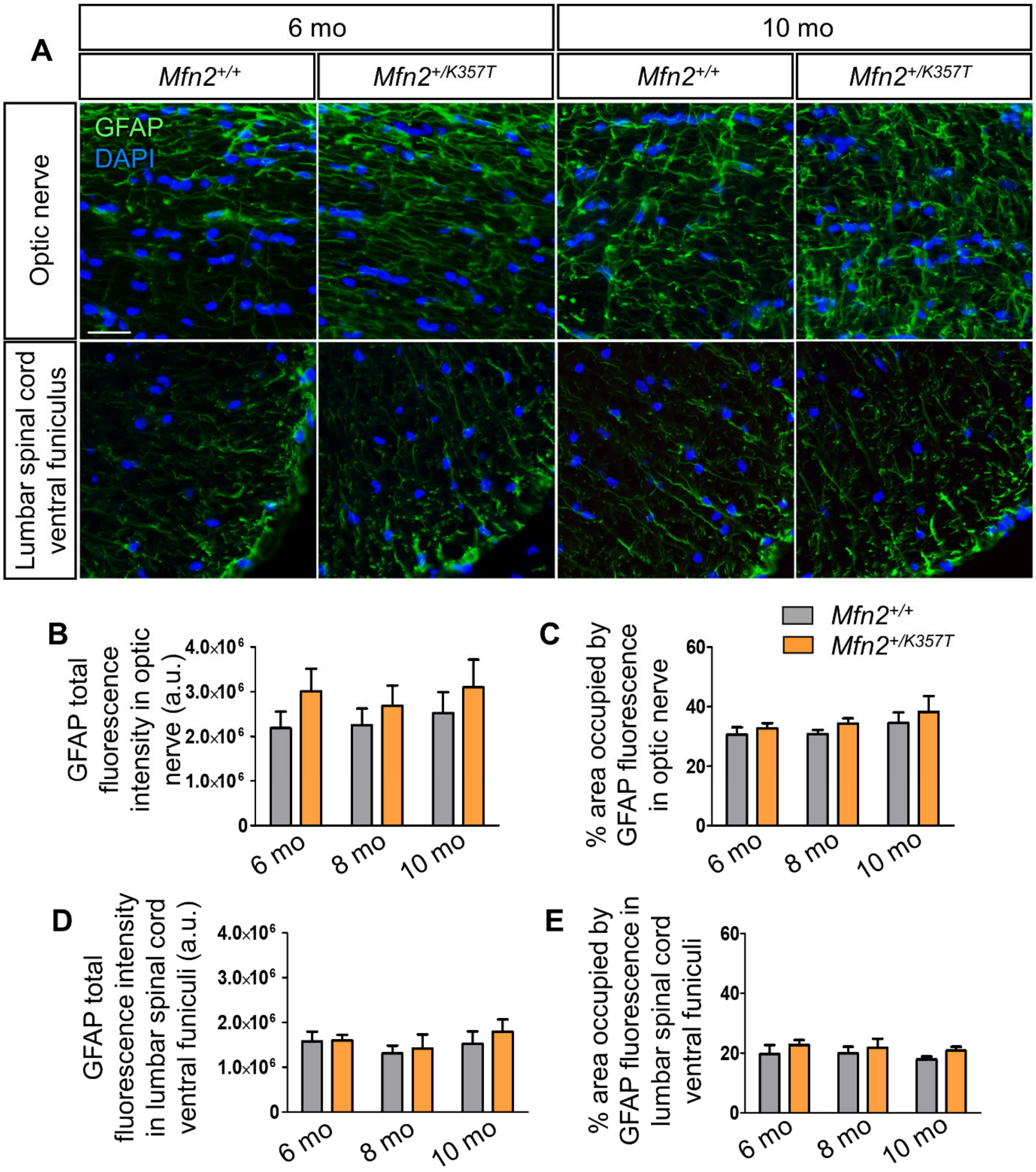

Figure S5. Lack of significant astrogliosis in optic nerves and lumbar spinal cords of *Mfn2*$^{+/K357T}$ mice. (**A**): Representative immunofluorescence images of optic nerve longitudinal sections and lumbar spinal cord ventral funiculus cross sections from 6– and 10–mo–old *Mfn2*$^{+/+}$ and *Mfn2*$^{+/K357T}$ mice. Sections were stained for astrocyte marker GFAP (green), and counterstained with DAPI (blue) (scale bar = 30 μm). (**B**–**E**): Semi–quantitative analysis of GFAP total fluorescence intensity (**B**, **D**) and area (**C**, **E**) in optic nerve longitudinal sections and lumbar spinal cord ventral funiculi cross sections from 6–, 8–, and 10–mo–old *Mfn2*$^{+/+}$ and *Mfn2*$^{+/K357T}$ mice (n = 4–5 mice/genotype) as indicated, measured in 6 15 x 15 cm ROI obtained from two 40 X images per mouse (3 ROI/image). a.u., arbitrary units. Data are presented as mean ± SEM. Two–way ANOVA with post hoc Bonferroni test was used for all comparisons. No statistical significance was reached.

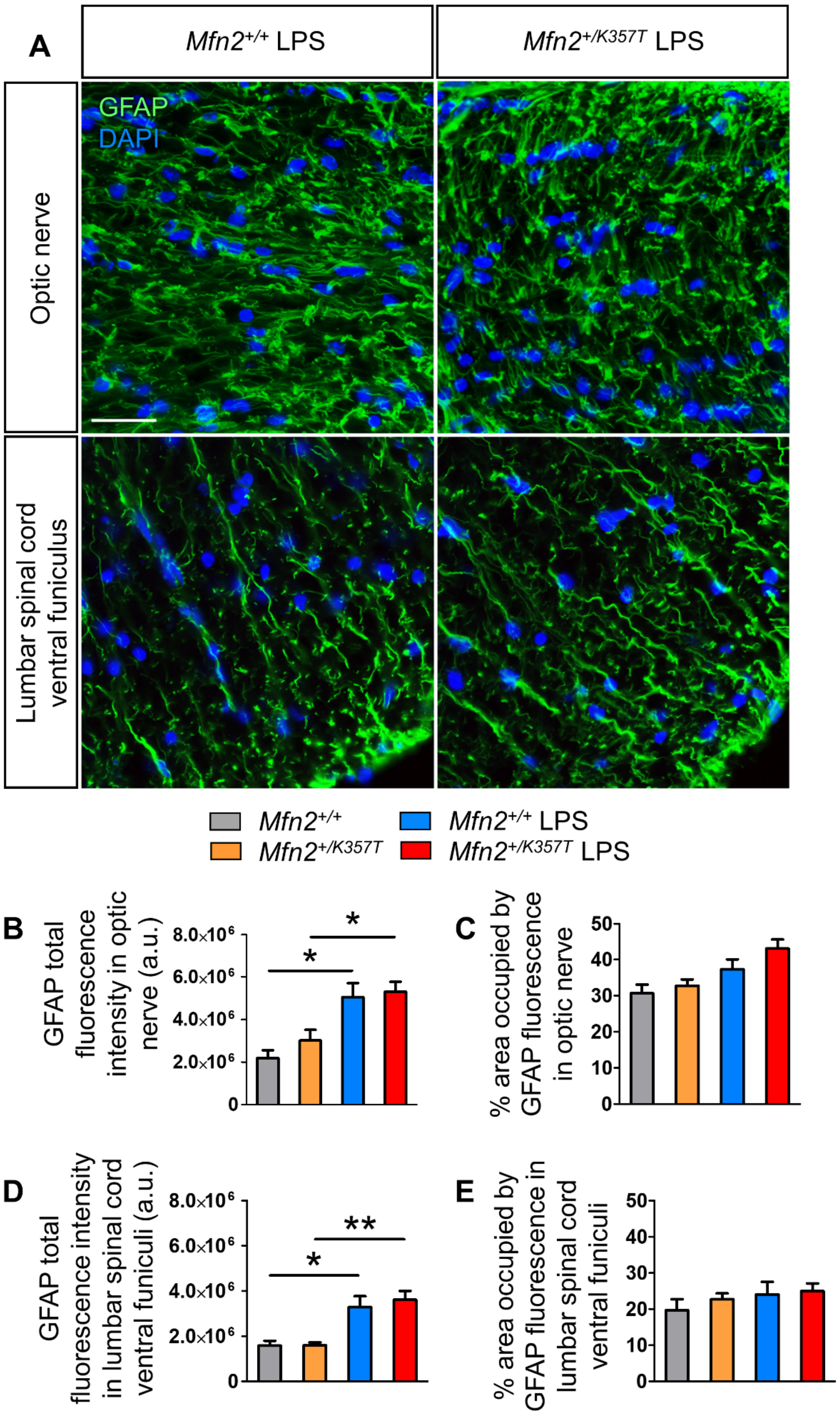
A
Mfn2+/+ LPS
Mfn2+/K357T LPS
GFAP
DAPI
Optic nerve
Lumbar spinal cord ventral funiculus
Mfn2+/+
Mfn2+/K357T
Mfn2+/+ LPS
Mfn2+/K357T LPS
B
GFAP total fluorescence intensity in optic nerve (a.u.)
8.0x10^6
6.0x10^6
4.0x10^6
2.0x10^6
0
*
*
C
% area occupied by GFAP fluorescence in optic nerve
50
40
30
20
10
0
D
GFAP total fluorescence intensity in lumbar spinal cord ventral funiculi (a.u.)
8.0x10^6
6.0x10^6
4.0x10^6
2.0x10^6
0
*
**
E
% area occupied by GFAP fluorescence in lumbar spinal cord ventral funiculi
50
40
30
20
10
0

Figure S6. Astrogliosis in optic nerves and lumbar spinal cords of LPS–injected *Mfn2*$^{+/+}$ and *Mfn2*$^{+/K357T}$ mice. (**A**): Representative immunofluorescence images of optic nerve longitudinal sections and lumbar spinal cord ventral funiculus cross sections from 6–mo–old *Mfn2*$^{+/+}$ and *Mfn2*$^{+/K357T}$ mice 96 h after LPS injection. Sections were stained against GFAP for astrocytes (green), and counterstained with DAPI (blue) (scale bar = 30 µm). (**B**–**E**): Semi–quantitative analysis of GFAP total fluorescence intensity (**B**, **D**) and area (**C**, **E**) in optic nerve longitudinal sections and lumbar spinal cord ventral funiculi cross sections at baseline conditions (without LPS injection; *n* = 4–5 mice/genotype) and 96 h after LPS injection (*n* = 6 mice/genotype), as indicated. GFAP total fluorescence intensity and area of occupancy were measured in 6 15 x 15 cm ROI obtained from two 40 X images per mouse (3 ROI/image). a.u., arbitrary units. Data are presented as mean ± SEM. One–way ANOVA with post hoc Tukey's HSD test was used for all comparisons: *p<0.05.

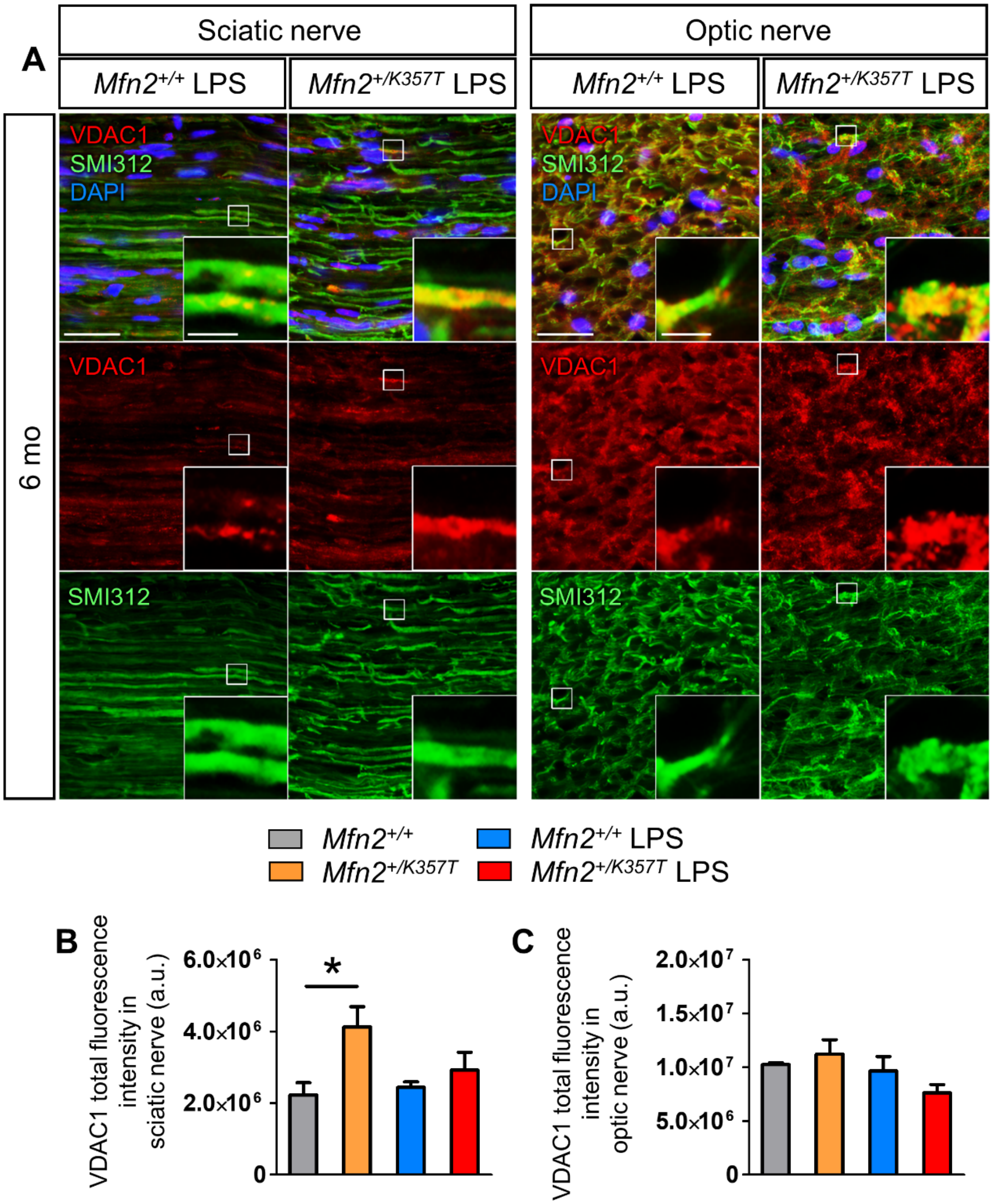

Figure S7. Mitochondrial localization in LPS–injected *Mfn2*$^{+/+}$ and *Mfn2*$^{+/K357T}$ mice. (**A**): Representative immunofluorescence images of sciatic and optic nerve longitudinal sections from 6–mo–old *Mfn2*$^{+/+}$ and *Mfn2*$^{+/K357T}$ mice 96 h after LPS injection. Sections were stained against VDAC1 for mitochondria (red), SMI312 for axons (green), and counterstained with DAPI (blue). Single and merged channels are shown. Magnified views (scale bar = 5 µm) from squared boxes in overviews (scale bar = 30 µm) are depicted in insets. Magnified views show the presence of VDAC1^{+} mitochondrial clusters in sciatic and optic nerve axons of *Mfn2*$^{+/K357T}$ LPS–treated mice. (**B**–**C**): Semi–quantitative analysis of VDAC1 total fluorescence intensity in sciatic (**B**) and optic nerve (**C**) longitudinal sections at baseline conditions (without LPS injection; *n* = 5 mice/genotype for sciatic nerves, *n* = 4 mice/genotype for optic nerves), and 96 h after LPS injection (*n* = 6 mice/genotype) measured in 6 15 x 15 cm ROI obtained from two 40 X images per mouse (3 ROI/image). a.u., arbitrary units. Data are presented as mean ± SEM. One–way ANOVA with post hoc Tukey's HSD test was used for all comparisons: *p<0.05.

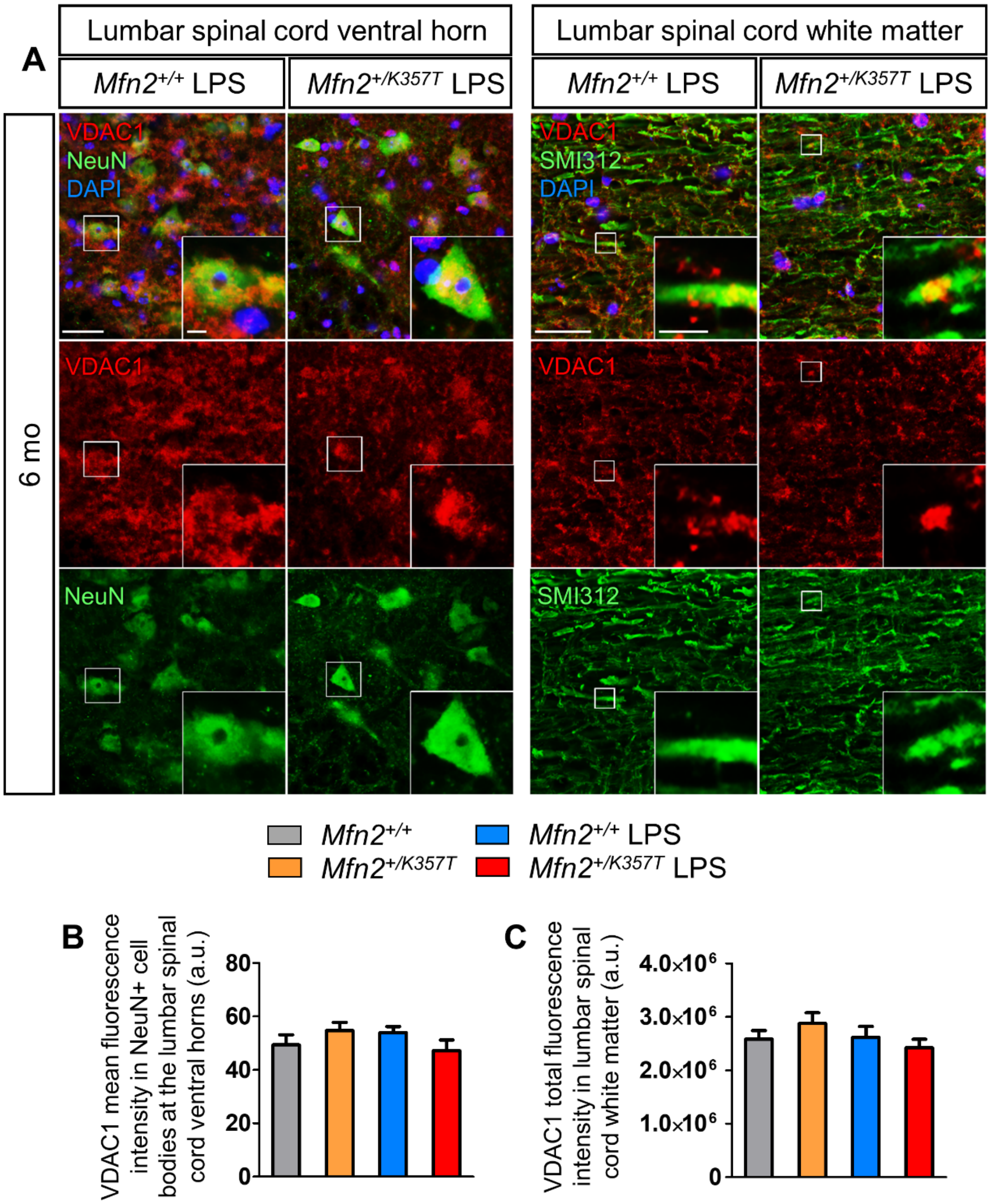

Figure S8. Mitochondrial localization in motor neuron cell bodies and white matter of LPS–injected *Mfn2*$^{+/+}$ and *Mfn2*$^{+/K357T}$ mice. (**A**): Representative immunofluorescence images of lumbar spinal cord motor neuron cell bodies in cross sections, and white matter longitudinal sections from 6–mo–old *Mfn2*$^{+/+}$ and *Mfn2*$^{+/K357T}$ mice 96 h after LPS injection. Sections were stained against VDAC1 for mitochondria (red), NeuN for neuronal cell bodies (green), SMI312 for axons (green), and counterstained with DAPI (blue). Single and merged channels are provided. Magnified views (scale bar = 5 µm) from squared boxes in overviews (scale bar = 30 µm) are depicted in insets. Magnified views show the presence of some VDAC1^{+} mitochondrial clusters in motor neuron cell bodies, and in white matter axons of *Mfn2*$^{+/K357T}$ LPS–treated mice. (**B**–**C**): Semi–quantitative analysis of VDAC1 mean fluorescence intensity in NeuN^{+} motor neuron cell bodies (**B**) and white matter (**C**) calculated from the VDAC1^{+} area overlapping with the NeuN^{+} area from n=10 NeuN^{+} cell bodies/mouse in B, or in 6 15 x 15 cm ROI obtained from two 40 X images per mouse (3 ROI/image) in C, at baseline conditions (n = 5 mice/genotype), and 96 h after LPS injection (n = 6 mice/genotype). a.u., arbitrary units. Data are presented as mean ± SEM. One–way ANOVA with post hoc Tukey's HSD test was used for all comparisons. No statistical significance was reached.
